# Cannulated screws versus dynamic hip screw versus hemiarthroplasty versus total hip arthroplasty in patients with displaced and non-displaced femoral neck fractures: a systematic review and frequentist network meta-analysis of 5703 patients

**DOI:** 10.1186/s13018-023-04114-8

**Published:** 2023-08-26

**Authors:** Nikolai Ramadanov, Katarzyna Jóźwiak, Michael Hauptmann, Philip Lazaru, Polina Marinova-Kichikova, Dobromir Dimitrov, Roland Becker

**Affiliations:** 1 Center of Orthopaedics and Traumatology, Brandenburg Medical School, University Hospital Brandenburg/Havel, Brandenburg an der Havel, Germany; 2 Institute of Biostatistics and Registry Research, Brandenburg Medical School Theodor Fontane, Neuruppin, Germany; 3 General and Visceral Surgery, Minimally Invasive Surgery and Coloproctology, St. Marien Hospital, Berlin, Germany; 4 Department of Surgical Propaedeutics, Faculty of Medicine, Medical University of Pleven, Pleven, Bulgaria; 5 Department of Surgical Diseases, Faculty of Medicine, Medical University of Pleven, Pleven, Bulgaria

**Keywords:** Cannulated screw, Dynamic hip screw, Hemiarthroplasty, Total hip arthroplasty, Hip replacement, Femoral neck fracture, Hip fracture, Network meta-analysis

## Abstract

**Background:**

Our aim was to determine the best operative procedure in human participants with a displaced or non-displaced femoral neck fracture comparing cannulated screw (CS) fixation, dynamic hip screw (DHS) fixation, hemiarthroplasty (HA), and total hip arthroplasty (THA) in terms of surgical and functional outcomes, reoperation and postoperative complications.

**Methods:**

We searched PubMed, The Cochrane Library, Clinical trials, CINAHL, and Embase for randomized controlled trials (RCTs) or quasi-RCTs up to 31 July 2022. A frequentist network meta-analysis was performed to assess the comparative effects of the four operative procedures, using fixed-effects and random-effects models. Mean differences (MDs) with 95% confidence intervals (CIs) were estimated for continuous variables and odds ratios (ORs) with 95% CIs were estimated for binary variables.

**Results:**

A total of 33 RCTs with 5703 patients were included in our network meta-analysis. CS fixation was best in terms of operation time (CS: MD = − 57.70, 95% CI − 72.78; − 42.62; DHS: MD = − 53.56, 95% CI − 76.17; − 30.95; HA: MD = − 20.90, 95% CI − 30.65; − 11.15; THA: MD = 1.00 reference) and intraoperative blood loss (CS: MD = − 3.67, 95% CI − 4.44; − 2.90; DHS: MD = − 3.20, 95% CI − 4.97; − 1.43; HA: MD = − 1.20, 95% CI − 1.73; − 0.67; THA: MD = 1.00 reference). In life quality and functional outcome, measured at different time points with EQ-5D and the Harris Hip Score (HHS), THA ranked first and HA second (e.g. EQ-5D 2 years postoperatively: CS: MD = − 0.20, 95% CI − 0.29; − 0.11; HA: MD = − 0.09, 95% CI − 0.17; − 0.02; THA: MD = 1.00 reference; HHS 2 years postoperatively: CS: MD = − 5.50, 95% CI − 9.98; − 1.03; DHS: MD = − 8.93, 95% CI − 15.08; − 2.78; HA: MD = − 3.65, 95% CI − 6.74; − 0.57; THA: MD = 1.00 reference). CS fixation had the highest reoperation risk, followed by DHS fixation, HA, and THA (CS: OR = 9.98, 95% CI 4.60; 21.63; DHS: OR = 5.07, 95% CI 2.15; 11.96; HA: OR = 1.60, 95% CI 0.89; 2.89; THA: OR = 1.00 reference).

**Conclusion:**

In our cohort of patients with displaced and non-displaced femoral neck fractures, HHS, EQ-5D, and reoperation risk showed an advantage of THA and HA compared with CS and DHS fixation. Based on these findings, we recommend that hip arthroplasty should be preferred and internal fixation of femoral neck fractures should only be considered in individual cases. Level of evidence I: a systematic review of randomized controlled trials.

*Trial registration*: PROSPERO on 10 August 2022 (CRD42022350293).

**Supplementary Information:**

The online version contains supplementary material available at 10.1186/s13018-023-04114-8.

## Introduction

One of the most common fractures in elderly patients is the femoral neck fracture [1]. It is associated with a high rate of morbidity and mortality in the elderly population [2]. The most common classifications used to evaluate femoral neck fractures are the Garden [3] and Pauwels [4] classifications. According to Pauwels, fractures are assessed from a mechanical point of view taking into account the orientation of the fracture line, which has an effect on the shear force and varus stress and thus the risk of fracture displacement. The Garden classification provides information on the degree of dislocation, and it is often used in decision-making for the preferred treatment of femoral neck fractures [5]. Garden I and II are defined as non-displaced (or minimally displaced), while Garden III and IV are defined as displaced femoral neck fractures [3]. There is a widespread agreement that displaced fractures should be treated with hip arthroplasty, while non-displaced fractures should be treated with internal fixation to preserve the femoral head [6, 7]. Deviations from this generally accepted procedure are not uncommon in individual cases. In recent years, several studies have shown that head preservation with internal fixation in elderly patients with displaced and non-displaced femoral neck fractures is associated with a high risk of reoperation, implant-related complications such as avascular necrosis of the femoral head, and nonunion [6–9]. Recent meta-analyses suggested that hip arthroplasty should also be performed in patients with non-displaced femoral neck fractures, as internal fixation was associated with a risk of poor clinical outcomes [6, 8]. On the other hand, numerous meta-analyses already highlighted some disadvantages of hemiarthroplasty (HA) and total hip arthroplasty (THA) in the treatment of patients with femoral neck fractures [10–14]. However, the network meta-analysis by Zhang et al. [15] was the first study that ranked the best operative procedure for femoral neck fractures. This 2017 network meta-analysis compared the outcomes of 7 operative procedures for displaced femoral neck fractures and showed that internal fixation had the highest, unipolar cemented HA had the lowest reoperation incidence; uncemented THA had the highest displacement incidence; and bipolar uncemented HA had the lowest infection incidence. This network meta-analysis focused on differentiating between cemented and uncemented implants, but, unfortunately, it did not differentiate between the type of internal fixation: cannulated screws (CS) or dynamic (sliding) hip screw (DHS). This is a serious limitation as the current literature has shown that DHS was superior to the CS for internal fixation of femoral neck fractures [16, 17]. Furthermore, the study by Zhang et al. [15] is limited to displaced femoral neck fractures. Therefore, there is an urgent need to address these limitations.

We formulated the following PICO (Population, Intervention, Control, and Outcomes) question: In human participants with a displaced or non-displaced femoral neck fracture, what is the best and worst operative procedure among CS fixation, DHS fixation, HA, and THA in terms of surgical and functional outcomes, reoperation and postoperative complications?

## Methods

The study protocol was registered in PROSPERO on 10 August 2022 (CRD42022350293). We adhered to the PRISMA Extension Statement for Reporting of Systematic Reviews Incorporating Network Meta-analyses of Health Care Interventions as the basis for the methodology and presentation of the data [18]. The PRISMA Checklist is available in Additional file 1. It must be taken into account that the group of authors of the present study has some experience in the field of meta-analyses and THAs. Similarities between all meta-analyses can only be attributed to the use of the same high-quality methods.

### Data sources and search strategies

We searched the following databases for randomized controlled trials (RCTs) or quasi-RCTs up to 31 July 2022: PubMed, The Cochrane Library, Clinical trials, CINAHL, and Embase. We developed a BOOLEAN search strategy, adapted to the syntax of the databases searched, using the following MeSH terms: ‘femoral neck fracture’, ‘displaced’, ‘undisplaced’, ‘non-displaced’, ‘nondisplaced’, ‘Garden’, ‘internal fixation’, ‘cannulated screws’, ‘sliding hip screw’, ‘dynamic hip screw’, ‘hemiarthroplasty’, ‘total hip arthroplasty’, ‘THA’, ‘THR’, ‘hip arthroplasty’, ‘hip replacement’. We additionally checked the reference list of related meta-analyses for relevant records. We did not review grey literature. There were no restrictions to publication language. We did not include RCTs that were older than 30 years.

### Screening and selection of RCTs

We searched for titles, abstracts, and finally full-text articles according to our inclusion criteria. RCTs were included on the basis of consensus between two reviewers (NR, PL). The RCT selection process was described in a flowchart diagram.

#### Inclusion criteria

The types of studies included were only RCTs. The types of participants included were only human participants with a displaced or non-displaced femoral neck fracture. The types of interventions included were the following: cannulated screw (CS) fixation, dynamic (sliding) hip screw (DHS) fixation with or without anti-rotation screws, hemiarthroplasty (HA), and total hip arthroplasty (THA). The types of outcome measures included were: operation time, intraoperative blood loss, EQ-5D [19], Harris Hip Score (HHS) [20], hospital stay, reoperation, mortality, and postoperative complications such as: deep vein thrombosis, hematoma, infection, intraoperative fracture, failure, avascular necrosis of the femoral head (ANFH), dislocation, and nonunion. “Failure” was defined as different types of osteosynthesis failure and loosening, as well as different types of prosthesis failure and loosening. There were no exclusion criteria regarding age and comorbidities of the participants.

### Statistical analysis

#### Data extraction and quality evaluation

Relevant data on RCT characteristics, methods, quality assessment, participant characteristics, operational details, relevant outcomes, and relevant additional information were extracted by two reviewers [NR, PL] independently from each other. The raw data extraction sheet is available in Additional file 2. The agreement between the reviewers was assessed using the Cohen's Kappa coefficient (κ). If the RCTs provided different information on intention to treat (ITT) and per protocol analysis, we adhered to the numbers from the ITT analysis. We compared the characteristics of the patient cohort between the 4 operative procedures, using Kruskal–Wallis tests and a significance level of 5%. Risk of bias was assessed using the Cochrane’s Risk of Bias 2 tool [21].

### Direct and indirect comparisons: network meta-analysis

A pairwise and network meta-analysis was performed to simultaneously assess the comparative effects of the four operations: CS fixation, DHS fixation, HA, and THA. All analyses were performed using fixed-effects and random-effects models estimated with the frequentist approach and consistency assumption. However, we only interpreted the results of the random-effects model as we believe that they can be generalized beyond the included studies. Mean differences (MDs) with 95% confidence intervals (CIs) were estimated for continuous variables and odds ratios (ORs) with 95% CIs were estimated for binary variables. In the case of a global and local validation of the consistency assumption, we created a net heat plot to decompose the between-design heterogeneity component into the contribution for each study design. Problematic RCTs were then removed and a network meta-analysis model was repeated. Two-arm comparisons from multi-arm RCTs were included in analyses with adjusted standard errors to account for the fact that comparisons within multi-arm RCTs were correlated. Between-study variance was estimated using the DerSimonian–Laird method, while heterogeneity was assessed using the Cochrane *Q* statistic test and the Higgins *I*2 test. Treatment effects together with the 95% CIs were presented on forest plots for separate pairwise comparisons evaluated in the individual studies and for comparison with THA or CS based on the network meta-analysis. The overall effect based on the network meta-analysis included the effect of the direct and indirect comparisons. In addition, we ranked the operations based on the cumulative probabilities for the highest to the lowest priority operations using the surface under the cumulative ranking curve (SUCRA) values obtained with 1000 simulations [22]. Analyses per outcome were performed for all RCTs together and included only RCTs with patients with displaced femoral neck fractures. All statistical analyses were performed using netmeta and metaphor packages in the R software version 4.2.0 [23].

### Missing data

Missing information on the standard deviation was imputed using a pooled value of all reported standard deviations calculated for each operation separately. If there was only one RCT for a given operation and it had a missing value, we replaced the standard deviation with a pooled value of all reported standard deviations calculated for all operations together. These imputed values were used for the main analyses [24]. Two sensitivity analyses were performed: (1) if standard deviation was not reported but the minimum and maximum values were given, an approximate standard deviation was obtained using the formula (max–min)/4 and (2) only RCTs that reported complete information were included.

## Results

**Fig. 1:**
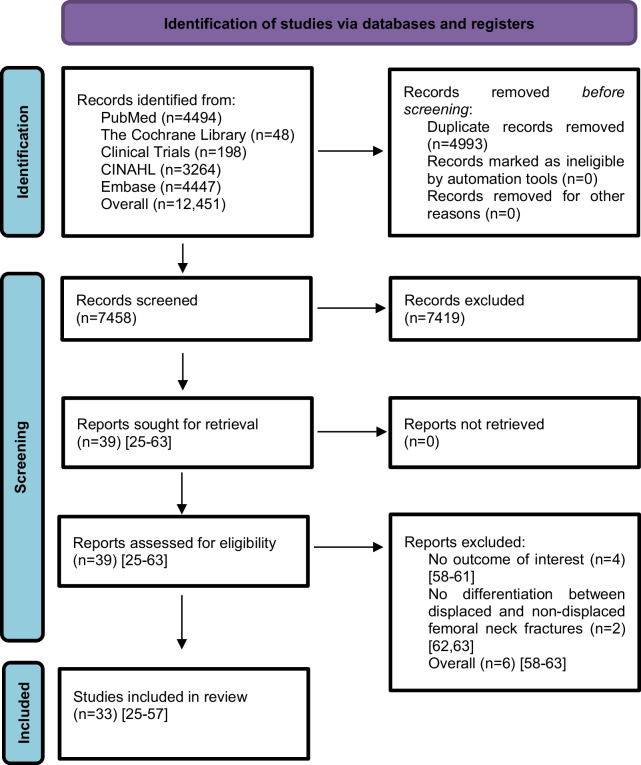
Flow chart of the article selection process

**Fig. 2:**
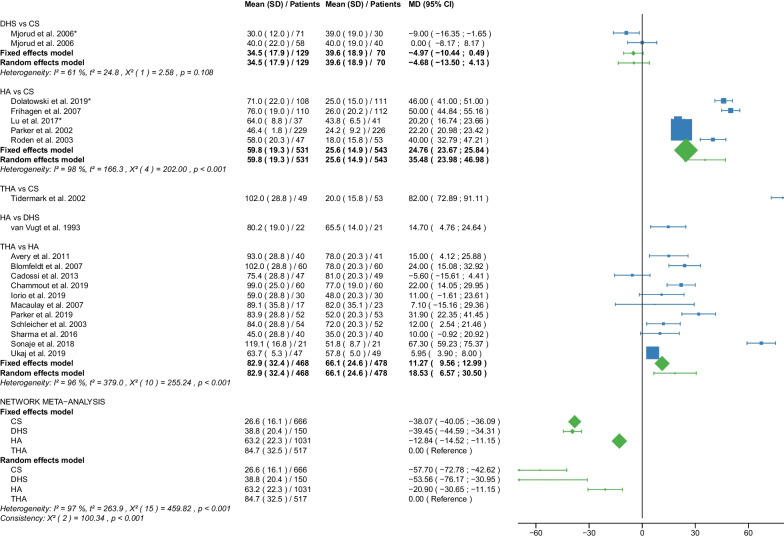
Forest plot of operation time. CS, cannulated screw; DHS, dynamic hip screw; HA, hemiarthroplasty; THA, total hip arthroplasty; SD, standard deviation; MD, mean difference; CI, confidence interval; *RCT with non-displaced fractures only

**Fig. 3:**
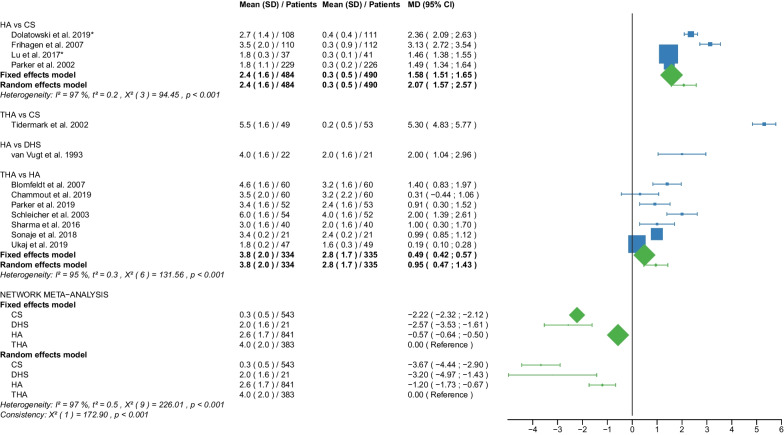
Forest plot of intraoperative blood loss.Results are shown for a unit of 100 ml. CS, cannulated screw; DHS, dynamic hip screw; HA, hemiarthroplasty; THA, total hip arthroplasty; SD, standard deviation; MD, mean difference; CI, confidence interval; *RCT with non-displaced fractures only

**Fig. 4:**
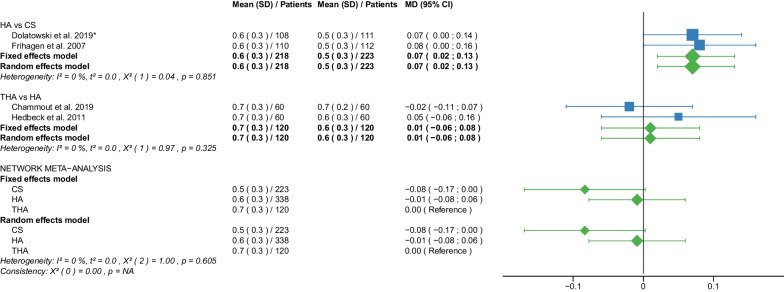
Forest plot of EQ 5D 3–4 months postoperatively. CS, cannulated screw; HA, hemiarthroplasty; THA, total hip arthroplasty; SD, standard deviation; MD, mean difference; CI, confidence interval; *RCT with non-displaced fractures only

**Fig. 5:**
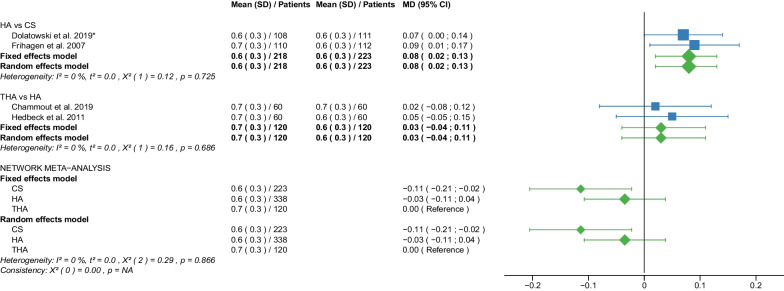
Forest plot of EQ 5D 12 months postoperatively. CS, cannulated screw; HA, hemiarthroplasty; THA, total hip arthroplasty; SD, standard deviation; MD, mean difference; CI, confidence interval; *RCT with non-displaced fractures only

**Fig. 6:**
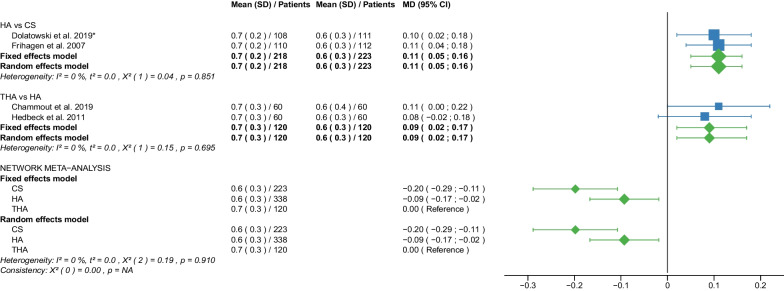
Forest plot of EQ 5D 2 years postoperatively. CS, cannulated screw; HA, hemiarthroplasty; THA, total hip arthroplasty; SD, standard deviation; MD, mean difference; CI, confidence interval; *RCT with non-displaced fractures only

**Fig. 7:**
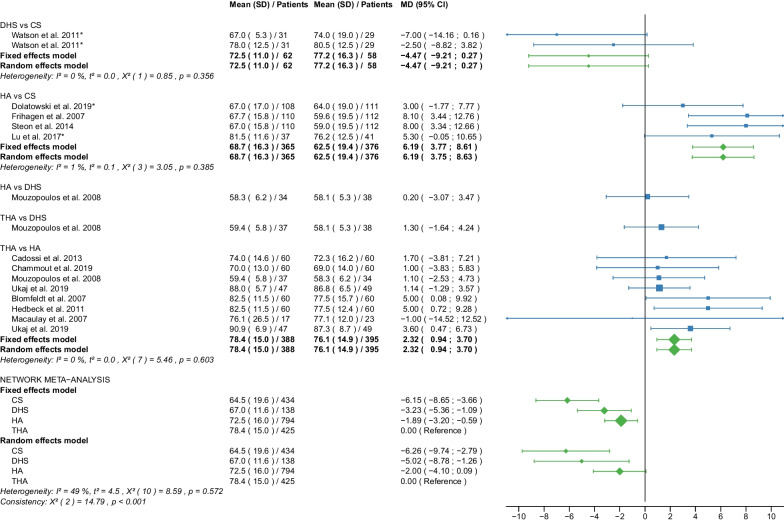
Forest plot of Harris Hip Score ≤ 6 months postoperatively. CS, cannulated screw; DHS, dynamic hip screw; HA, hemiarthroplasty; THA, total hip arthroplasty; SD, standard deviation; MD, mean difference; CI, confidence interval; *RCT with non-displaced fractures only

**Fig. 8:**
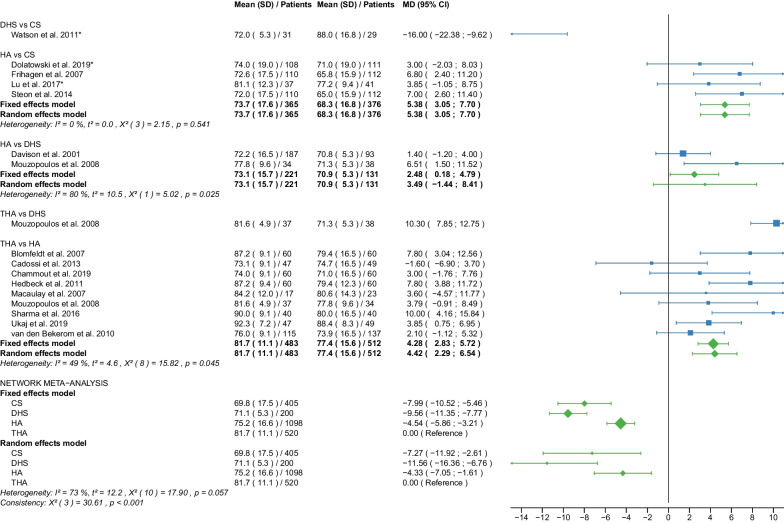
Forest plot of Harris Hip Score 12 months postoperatively. CS, cannulated screw; DHS, dynamic hip screw; HA, hemiarthroplasty; THA, total hip arthroplasty; SD, standard deviation; MD, mean difference; CI, confidence interval; *RCT with non-displaced fractures only

**Fig. 9:**
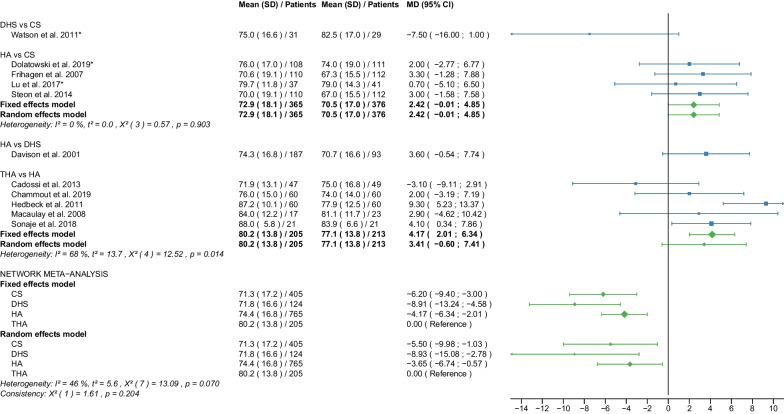
Forest plot of Harris Hip Score 2 years postoperatively. CS, cannulated screw; DHS, dynamic hip screw; HA, hemiarthroplasty; THA, total hip arthroplasty; SD, standard deviation; MD, mean difference; CI, confidence interval; *RCT with non-displaced fractures only

**Fig. 10:**
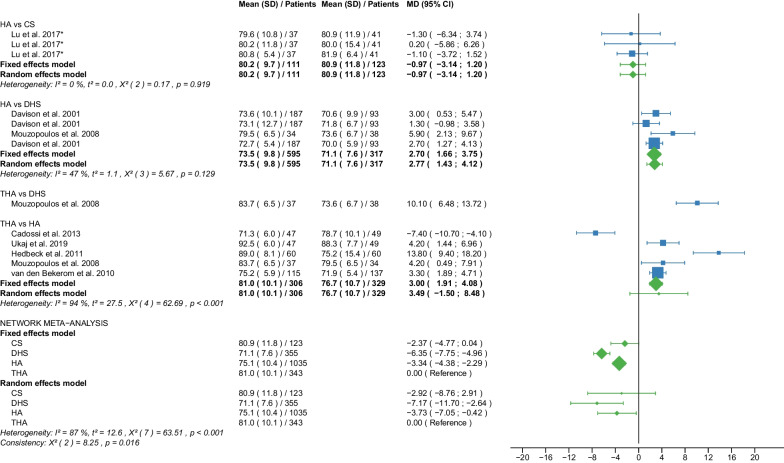
Forest plot of Harris Hip Score 3–5 years postoperatively. CS, cannulated screw; DHS, dynamic hip screw; HA, hemiarthroplasty; THA, total hip arthroplasty; SD, standard deviation; MD, mean difference; CI, confidence interval; *RCT with non-displaced fractures only

**Fig. 11:**
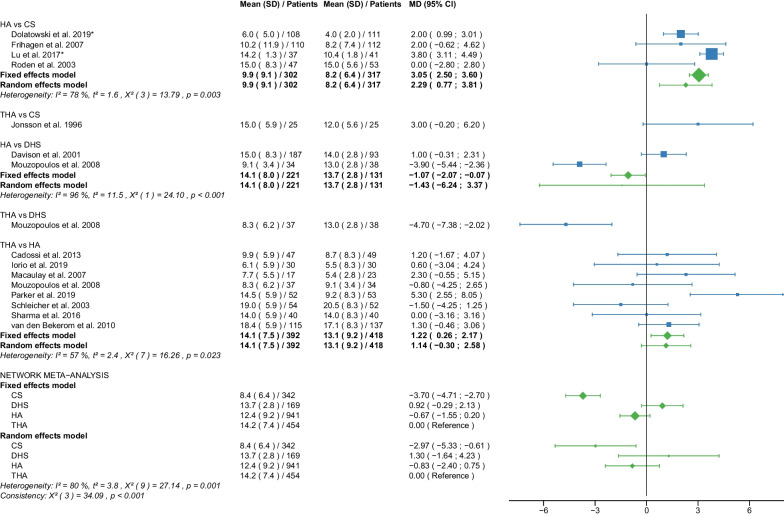
Forest plot of hospital stay. CS, cannulated screw; DHS, dynamic hip screw; HA, hemiarthroplasty; THA, total hip arthroplasty; SD, standard deviation; MD, mean difference; CI, confidence interval; *RCT with non-displaced fractures only

**Fig. 12:**
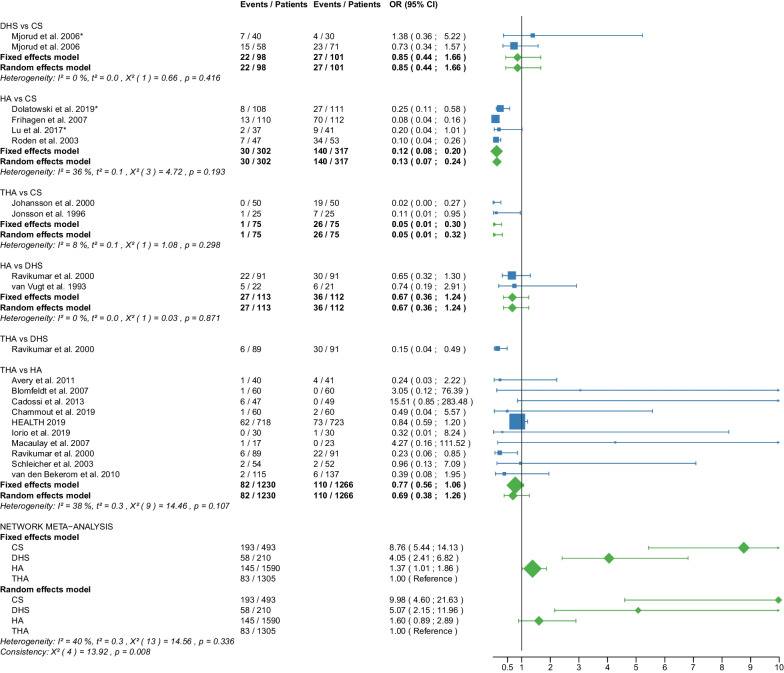
Forest plot of reoperation. CS, cannulated screw; DHS, dynamic hip screw; HA, hemiarthroplasty; THA, total hip arthroplasty; OR, odds ratio; CI, confidence interval; *RCT with non-displaced fractures only

**Fig. 13:**
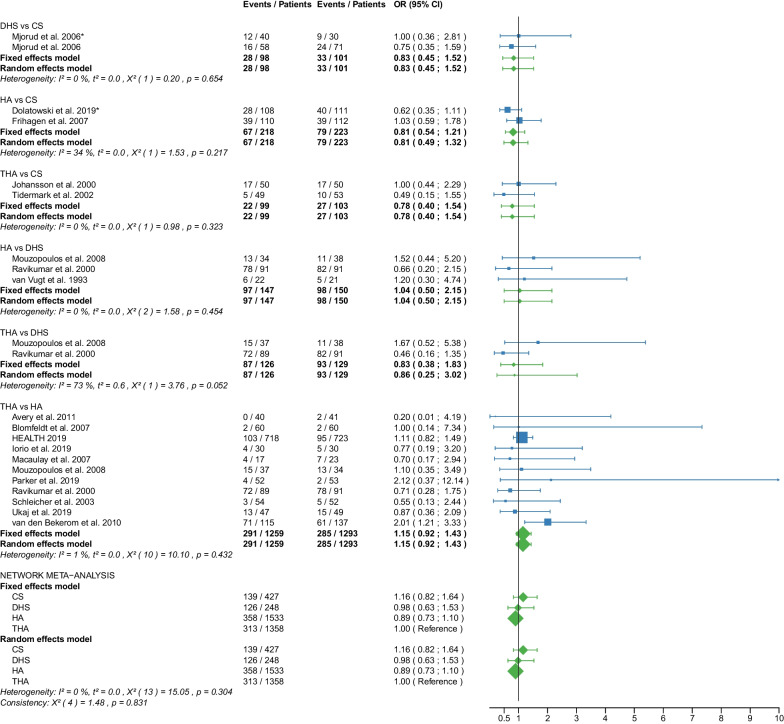
Forest plot of mortality. CS, cannulated screw; DHS, dynamic hip screw; HA, hemiarthroplasty; THA, total hip arthroplasty; OR, odds ratio; CI, confidence interval; *RCT with non-displaced fractures only

**Fig. 14:**
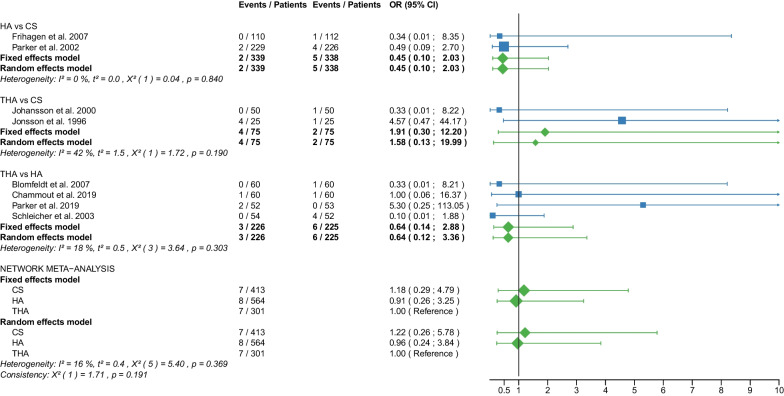
Forest plot of deep vein thrombosis. CS, cannulated screw; HA, hemiarthroplasty; THA, total hip arthroplasty; OR, odds ratio; CI, confidence interval

**Fig. 15:**
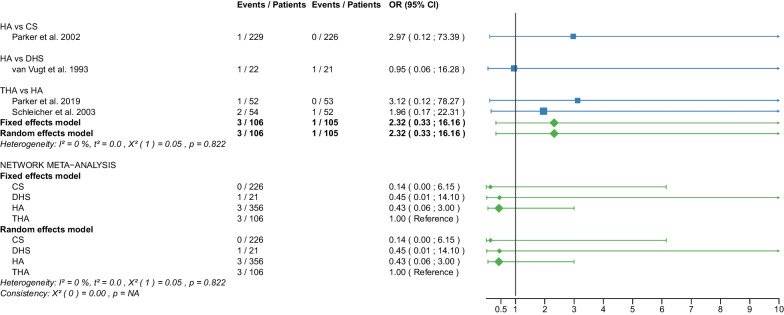
Forest plot of hematoma. CS, cannulated screw; DHS, dynamic hip screw; HA, hemiarthroplasty; THA, total hip arthroplasty; OR, odds ratio; CI, confidence interval

**Fig. 16:**
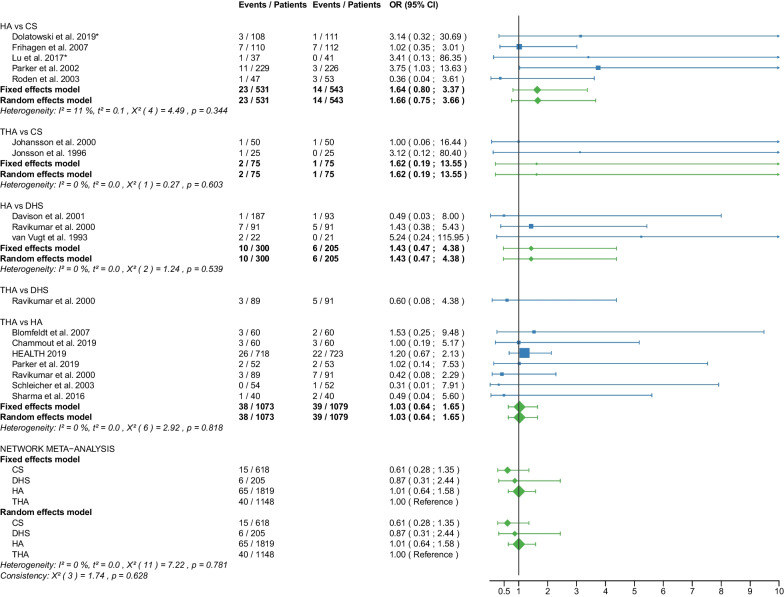
Forest plot of infection. CS, cannulated screw; DHS, dynamic hip screw; HA, hemiarthroplasty; THA, total hip arthroplasty; OR, odds ratio; CI, confidence interval; *RCT with non-displaced fractures only

**Fig. 17:**
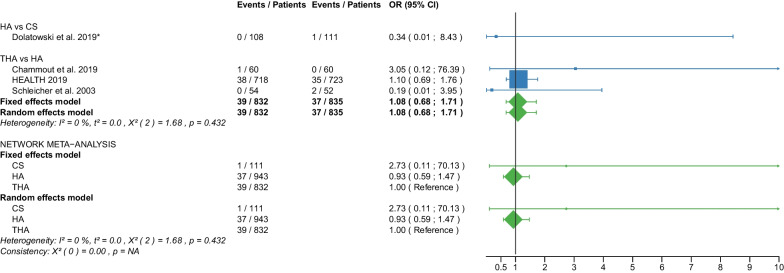
Forest plot of intraoperative fracture. CS, cannulated screw; HA, hemiarthroplasty; THA, total hip arthroplasty; OR, odds ratio; CI, confidence interval; *RCT with non-displaced fractures only

**Fig. 18:**
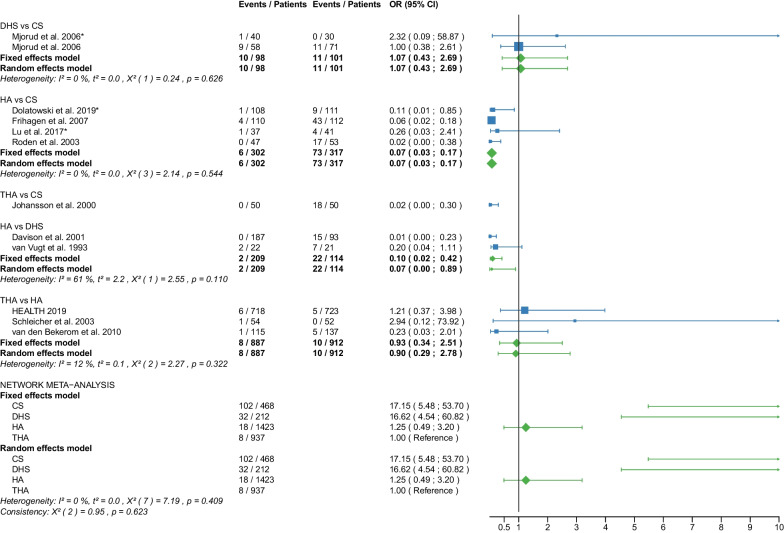
Forest plot of failure. CS, cannulated screw; DHS, dynamic hip screw; HA, hemiarthroplasty; THA, total hip arthroplasty; OR, odds ratio; CI, confidence interval; *RCT with non-displaced fractures only

**Fig. 19:**
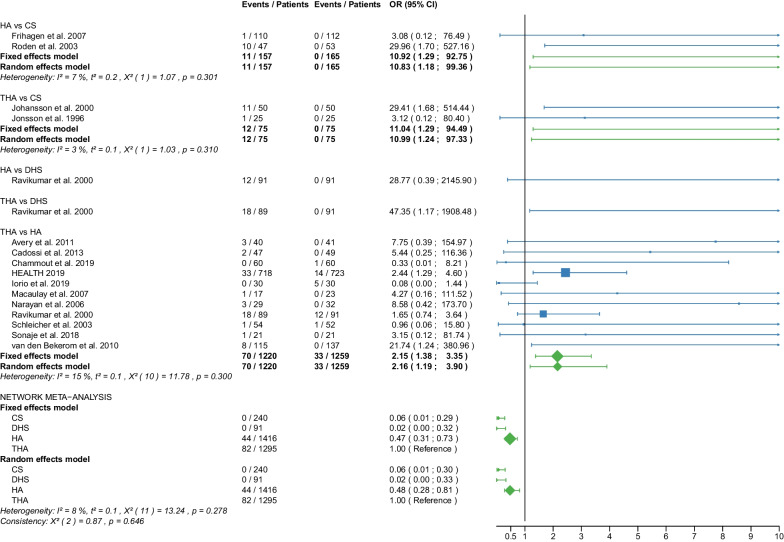
Forest plot of dislocation. CS, cannulated screw; DHS, dynamic hip screw; HA, hemiarthroplasty; THA, total hip arthroplasty; OR, odds ratio; CI, confidence interval

**Fig. 20:**
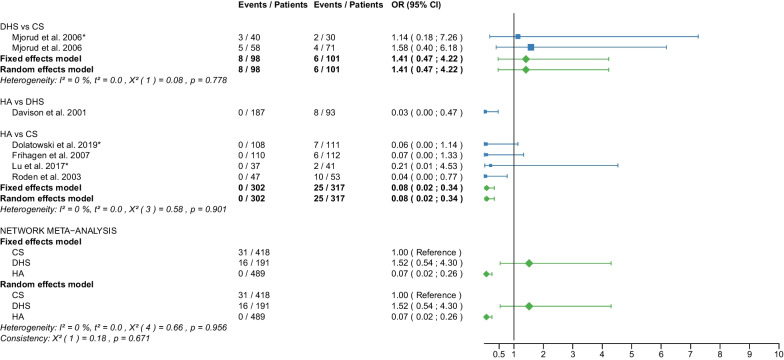
Forest plot of ANFH. CS, cannulated screw; DHS, dynamic hip screw; HA, hemiarthroplasty; OR, odds ratio; CI, confidence interval; *RCT with non-displaced fractures only

**Fig. 21:**
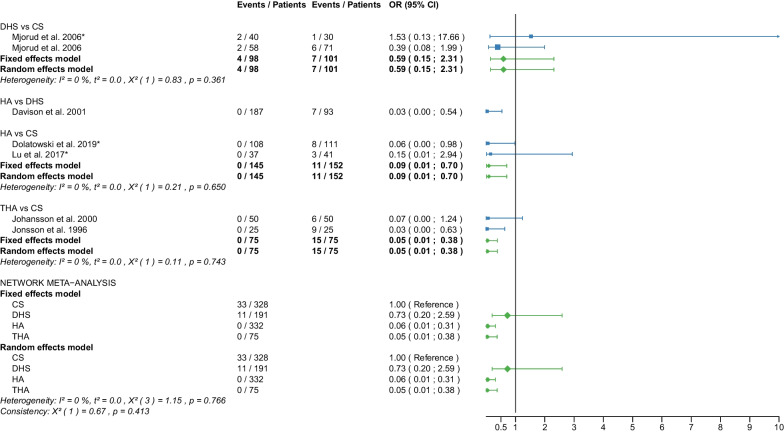
Forest plot of nonunion. CS, cannulated screw; DHS, dynamic hip screw; HA, hemiarthroplasty; THA, total hip arthroplasty; OR, odds ratio; CI, confidence interval; *RCT with non-displaced fractures only

The study selection process is shown in a flowchart (Fig. 1). After removal of duplicates and screening of titles and abstracts with a high inter-reviewer agreement (*κ* = 0.98), 39 RCTs were assessed for eligibility [25–63]. After the second screening procedure by full-text analysis (*κ* = 1.0), 6 RCTs were excluded for the following reasons: 4 of these RCTs did not report any of the outcomes of interest [58–61], 2 RCTs did not differentiate between displaced and non-displaced femoral neck fractures [62, 63]. A total of 33 RCTs [25–57] with 5703 patients were included in our network meta-analysis. Of these patients, 5276 (92%) had a displaced femoral neck fracture, and 427 (8%) had a non-displaced femoral neck fracture. The fractures were operated as follows: CS fixation in 913 (16%) patients, DHS fixation in 372 (6.5%) patients, HA in 2606 (46%) patients, and THA in 1812 (31.5%) patients. Clinical characteristics for age, sex, time to surgery and follow-up period (Tables 1, 2) showed no significant differences between the 4 operative procedures. On average, patients were 79 years of age (range: 59–86), 26% of them were males, their time to surgery was 43 h and they were followed up for 46.3 months. On average, the patients of the CS fixation group were 82 years of age (range: 77–86), 24% were males, their time to surgery was 30 h and they were followed-up for 27 months. On average, the patients of the DHS fixation group were 77 years of age (range: 73–81), 26% were males, their time to surgery was 37 h and they were followed up for 53 months. On average, the patients of the HA group were 78 years of age (range: 63–86), 26% were males, their time to surgery was 46 h and they were followed up for 48 months. On average, the patients of the THA group were 78 years of age (range: 59–85), 27% were males, their time to surgery was 54 h and they were followed up for 51 months. Further details on the patient cohort and the included RCTs are given in Table 1. It is important to notice that two RCTs [42, 46] did not compare 2 operative procedures, but 3 operative procedures: DHS fixation, HA, and THA. Another RCT [30] compared CS fixation with two different types of cemented HA. The distinction between the two types of cemented HA was not relevant to our network meta-analysis and the data from the two HAs were pooled (Table 2). Another RCT [41] investigating CS fixation and DHS fixation provided data that included displaced and non-displaced fractures. Accordingly, 4 different operative procedures (CS fixation for displaced fractures, CS fixation for non-displaced fractures, DHS fixation for displaced fractures, and DHS fixation for non-displaced fractures) were included in the network meta-analysis of this study [41]. RCTs that included non-displaced fractures [31, 38, 41, 57] are marked accordingly in the forest plots. The assessment of the risk of bias is shown in Table 3. Some RCTs had a high risk of bias [25–28, 31, 33–35, 44–50, 52, 55, 56]. One of them is more likely to be considered a quasi-RCT [48]. The statistical heterogeneity of all outcomes measured is shown in Figs. 2, 3, 4, 5, 6, 7, 8, 9, 10, 11, 12, 13, 14, 15, 16, 17, 18, 19, 20 and 21.

**Table 1:** Patient and RCT details

RCT	Year	Country of origin	Operation	Approach	Cement usage, *N* (%)	Femoral neck fracture	Patients, *N*	Age, years	Age, range or SD	Male sex, *N* (%)	Time to surgery, hours	Time to surgery, range or SD	Follow up period, months	Follow up period, range or SD	Outcome reported
Avery PP et al. [25]	2011	United Kingdom	HA	L	41 (100)	D	41	76	66–86	9 (22)	NR	NR	103.2	86.4–120	1,11,12,18,21
			THA	L	40 (100)	D	40	74	63–86	8 (20)	NR	NR	106	86.4–122.4	
Baker RP et al. [26]	2006	United Kingdom	HA	L	41 (100)	D	41	76	66–86	9 (22)	NR	NR	36	NR	1,11,12,18 (identical with Avery PP et al. [25])
			THA	L	40 (100)	D	40	74	63–86	8 (20)	NR	NR	36	NR	
Blomfeldt et al. [27]	2007	Sweden	HA	AL	60 (100)	D	60	81	70–89	6 (10)	NR	NR	12	NR	1,2,6,7,11, 12,13,15
			THA	AL	60 (100)	D	60	81	70–90	13 (22)	NR	NR	12	NR	
Cadossi M et al. [28]	2013	Italy	HA	L	33 (67)	D	49	84	73–98	13 (27)	86.4	24–168	30.1	23–50	1,6,7,8,9, 10,11,18
			THA	L	0 (0)	D	47	82	71–96	8 (17)	69.6	24–192	28.6	22–52	
Chammout G et al. [29]	2019	Sweden	HA	L	60 (100)	D	60	86	4	15 (25)	NR	NR	24	NR	1,2,3,4,5, 6,7,8,11,13, 15,16,18
			THA	L	60	D	60	85	4	15 (25)	NR	NR	24	NR	
Davison JN et al. [30]	2001	United Kingdom	DHS	NA	0 (0)	D	93	73	70–77	23 (25)	48	24–48	24	NR	7,8,9,10,15, 17,19,20
			HA	L	187 (100)	D	187	75	71–78	44 (24)	48	24–72	24	NR	
Dolatowski FC et al. [31]	2019	Norway	CS	NA	NR	N	111	83	7	27 (24)	29	21	24	NR	1,2,3,4,5, 6,7,8,10,11, 12,15,16,17, 19,20
			HA	L	NR	N	108	83	7	35 (32)	28	19	24	NR	
Frihagen F et al. [32]	2007	Norway	CS	NA	0 (0)	D	112	83	8	25 (22)	25.3	15.3	24	NR	1,2,3,4,5, 6,7,8,10,11, 12,13,15,17, 18,19
			HA	L	110 (100)	D	110	83	7	32 (29)	31.4	22.3	24	NR	
HEALTH [33]	2019	USA	HA	NR	NR	D	723	79	9	223 (31)	NR	NR	24	NR	11,12,15,16, 17,18
			THA	NR	NR	D	718	79	8	208 (29)	NR	NR	24	NR	
Hedbeck CJ et al. [34]	2011	Sweden	HA	AL	60 (100)	D	60	81	5	6 (10)	NR	NR	48	NR	3,4,5,6,7, 8,9
			THA	AL	60 (100)	D	60	81	5	13 (22)	NR	NR	48	NR	
Iorio R et al. [35]	2019	Italy	HA	L	0 (0)	D	30	83	3	13 (43)	51	12–72	12	NR	1,10,11,12, 18
			THA	L	0 (0)	D	30	82	4	12 (40)	59	16–68	12	NR	
Johansson T et al. [36]	2000	Sweden	CS	NA	50 (100)	D	50	84	75–96	16 (32)	NR	NR	24	NR	11,12,13,15, 17,18,20
			THA	PL	50 (100)	D	50	84	75–101	10 (20)	NR	NR	24	NR	
Jonsson B et al. [37]	1996	Sweden	CS	NA	NR	D	25	79	70–89	NR	NR	NR	24	NR	10,11,13,15, 18,20
			THA	L	NR	D	25	80	67–89	NR	NR	NR	24	NR	
Lu Q et al. [38]	2017	China	CS	NA	0 (0)	N	41	85	4	12 (29)	NR	NR	38.7	28.2	1,2,6,7,8, 9,10,11,15, 17,19,20
			HA	AL	37 (100)	N	37	86	5	8 (22)	NR	NR	38.7	28.2	
Macaulay W et al. [39]	2007	USA	HA	L,PL	NR	D	23	77	9	9 (39)	NR	NR	19	13–33	1,6,7,10,11, 12,18
			THA	L,PL	NR	D	17	82	7	10 (59)	NR	NR	19	13–33	
Macaulay W et al. [40]	2008	USA	HA	AL,PL,P	NR	D	23	77	9	9 (39)	NR	NR	24	NR	8
			THA	AL,PL,P	NR	D	17	82	9	10 (59)	NR	NR	24	NR	
Mjorud J et al. [41]	2006	Norway	CS	NA	NR	N	30	79	16	8 (27)	54	94	24	NR	1,11,12,17, 19,20
			CS	NA	NR	D	71	82	9	15 (21)	17	14	24	NR	
			DHS	NA	NR	N	40	79	13	6 (15)	37	74	24	NR	
			DHS	NA	NR	D	58	81	8	18 (31)	20	31	24	NR	
Mouzopoulos G et al. [42]	2008	Greece	DHS	NA	NR	D	38	75	5	12 (32)	44.2	5.2	48	NR	6,7,9,10,12
			HA	NR	NR	D	34	74	4	10 (29)	45.8	2.4	48	NR	
			THA	NR	NR	D	37	73	5	9 (14)	45.2	7.3	48	NR	
Narayan K et George T [43]	2006	India	HA	L	NR	D	32	63	6	NR	NR	NR	58.5	17.6	18
			THA	L	NR	D	29	59	6	NR	NR	NR	58.5	17.6	
Parker MJ et al. [44]	2002	United Kingdom	CS	NA	0 (0)	D	226	82	71–103	45 (20)	25	NR	36	NR	1,2,13,14, 15
			HA	AL	0 (0)	D	229	82	71–101	46 (20)	27.5	NR	36	NR	
Parker MJ et Cawley S [45]	2019	United Kingdom	HA	AL	53 (100)	D	53	77	60–89	8 (15)	NR	NR	35.5	NR	1,2,10,12, 13,14,15
			THA	AL	52 (100)	D	52	77	67–89	12 (23)	NR	NR	35.5	NR	
Ravikumar KJ et al. [46]	2000	United Kingdom	DHS	NA	0 (0)	D	91	NR	NR	27 (10)	NR	NR	156	NR	11,12,15,18
			HA	PL	0 (0)	D	91	NR	NR		NR	NR	156	NR	
			THA	PL	89 (100)	D	89	NR	NR		NR	NR	156	NR	
Roden M et al. [47]	2003	Sweden	CS	NA	0 (0)	D	53	81	70–96	16 (30)	NR	NR	NR	NR	1,10,11,15, 17,18,19
			HA	P	47 (100)	D	47	81	70–96	13 (28)	NR	NR	NR	NR	
Schleicher I et al. [48]	2003	Germany	HA	NR	NR	D	52	81	54–94	9 (17)	NR	NR	24	NR	1,2,10,11, 12,13,14,15, 16,17,18
			THA	NR	NR	D	54	81	46–94	7 (13)	NR	NR	24	NR	
Sharma V et al. [49]	2016	India	HA	P	NR	D	40	73	62–67	11 (28)	72	NR	NR	NR	1,2,7,10,15
			THA	P	NR	D	40	78	65–79	14 (35)	72	NR	NR	NR	
Sonaje JC et al. [50]	2018	India	HA	NR	21 (100)	D	21	65	61–73	6 (29)	NR	NR	24	NR	1,2,8,18
			THA	NR	21 (100)	D	21	66	60–74	7 (33)	NR	NR	24	NR	
Steon RO et al. [51]	2014	Norway	CS	NA	0 (0)	D	112	NR	NR	NR	NR	NR	NR	NR	6,7,8
			HA	L	110 (100)	D	110	NR	NR	NR	NR	NR	NR	NR	
Tidermark J et al. [52]	2002	Sweden	CS	NA	0 (0)	D	53	81	7	11 (21)	NR	NR	NR	NR	1,2,12
			THA	AL	0 (0)	D	49	79	5	9 (18)	NR	NR	NR	NR	
Tol MC et al. [53]	2017	Netherlands	HA	NR	NR	D	137	80	6	22 (16)	NR	NR	168	NR	11,18 (not meta-analysed)
			THA	NR	NR	D	115	82	6	25 (22)	NR	NR	168	NR	
Ukaj S et al. [54]	2019	Kosovo	HA	P	NR	D	49	78	5	33 (68)	NR	NR	NR	NR	1,2,6,7,9, 12
			THA	P	NR	D	47	78	5	23 (49)	NR	NR	NR	NR	
van den Bekerom MPJ et al. [55]	2010	Netherlands	HA	AL,PL	NR	D	137	80	70–94	22 (16)	24	0–10	120	NR	7,9,10,11, 12,17,18
			THA	AL,PL	NR	D	115	82	70–96	25 (22)	24	0–9	120	NR	
van Vugt AB et al. [56]	1993	Netherlands	DHS	NA	0 (0)	D	21	75	3	10 (48)	NR	NR	36	NR	1,2,11,12, 14,15,17
			HA	AL	22 (100)	D	22	76	3	8 (37)	NR	NR	36	NR	
Watson A et al. [57]	2011	Australia	CS	NA	0 (0)	N	29	77	53–93	5 (17)	NR	NR	24	NR	6,7,8
			DHS	NA	0 (0)	N	31	78	53–89	6 (19)	NR	NR	24	NR	

RCT, randomized controlled trial; CS, cannulated screw; DHS, dynamic hip screw; HA, hemiarthroplasty; THA, total hip arthroplasty; NA, not applicable; NR, not reported; L, lateral approach; AL, anterolateral approach; P, posterior; PL, posterolateral approach; D, displaced; N, non-displaced; *N*, number; SD, standard deviation; 1: Operation time; 2: Intraoperative blood loss; 3: EQ 5D 3–4 months postoperatively; 4: EQ 5D 12 months postoperatively; 5: EQ 5D 2 years postoperatively; 6: Harris Hip Score ≤ 6 months postoperatively; 7: Harris Hip Score 12 months postoperatively; 8: Harris Hip Score 2 years postoperatively; 9: Harris Hip Score 3–5 years postoperatively; 10: Hospital stay; 11: Reoperation; 12: Mortality; 13: Deep vein thrombosis; 14: Hematoma; 15: Infection; 16: Intraoperative fracture; 17: Loosening; 18: Dislocation; 19: ANFH; 20: Nonunion; 21: Acetabular cup erosion

**Table 2:** Clinical characteristics of the patient cohort

Patient characterics	Total	CS fixation	DHS fixation	HA	THA	*p* value
	Mean/median (interquantile1-interquantile3)					
Age (years)	78.7/80.3 (76.7–82.1)	81.6/82.0 (79.0–83.2)	76.9/76.6 (75.3- 79.0)	78.4/79.4 (75.8–82.4)	78.2/80.3 (77.1–82.0)	0.063
Male sex (%)	26.3/23.5 (19.5–31.0)	24.3/23.0 (21.0–29.0)	25.7/25.0 (15.0–32.0)	26.5/26.0 (17.0–31.0)	27.2/22.0 (20.0–33.0)	0.995
Time to surgery (hours)	42.8/44.2 (25.3–54.0)	30.1/25.3 (25.0–29.0)	37.3/40.6 (28.5–46.1)	46.0/45.8 (28.0–51.0)	54.0/59.0 (45.2–69.6)	0.283
Follow-up (months)	46.3/24.0 (24.0–48.0)	27.0/24.0 (24.0–24.0)	53.1/36.0 (24.0–60.0)	47.9/32.8 (24.0–48.0)	50.8/26.3 (24.0–53.3)	0.540

*P* values are obtained with Kruskal–Wallis test. CS, cannulated screw; DHS, dynamic hip screw; HA, hemiarthroplasty; THA, total hip arthroplasty

**Table 3:** Risk of bias assessment

	Random sequence generation (selection bias)	Allocation concealment (selection bias)	Blinding of participants and personnel (performance bias)	Blinding of outcome assessment (detection bias)	Incomplete outcome data (attrition bias)	Selective reporting (reporting bias)	Other bias
Avery et al. 2011	+	+	−	?	?	+	+
Baker et al. 2006	+	+	−	?	?	+	+
Blomfeldt et al.	+	+	−	+	+	+	?
Cadossi et al.	+	+	−	?	+	+	?
Chammout et al.	+	+	+	+	+	+	?
Davison et al.	+	?	+	+	+	?	?
Dolatowski et al.	+	+	−	+	+	+	+
Frihagen et al.	+	+	+	+	+	?	?
HEALTH	+	+	−	−	?	+	?
Hedbeck et al.	+	+	−	+	+	+	?
Iorio et al.	?	?	−	+	+	?	+
Johansson et al.	+	+	?	?	+	?	?
Jonsson et al.	+	+	?	?	+	?	?
Lu et al.	+	?	?	+	+	+	+
Macaulay et al. 2007	+	+	+	?	+	+	+
Macaulay et al. 2008	+	+	+	?	+	+	+
Mjorud et al.	+	+	?	?	+	+	?
Mouzopoulos et al.	?	?	?	+	+	+	?
Narayan et al.	+	+	?	?	+	+	?
Parker et al. 2002	+	+	−	+	+	+	?
Parker et al. 2019	+	+	−	+	+	+	?
Ravikumar et al.	−	?	?	?	+	+	?
Röden et al.	?	+	−	−	+	?	−
Schleicher et al.	?	?	−	−	?	?	−
Sharma et al.	+	+	−	?	+	+	?
Sonaje et al.	?	?	?	−	?	+	+
Stoen et al.	+	+	+	+	+	+	+
Tidermark et al.	?	+	−	−	+	?	−
Tol et al.	+	+	?	?	?	+	?
Ukaj et al.	+	+	+	?	+	+	?
Van der Bekerom et al.	+	+	−	−	+	+	+
Van Vugt et al.	?	?	−	−	−	?	−
Watson et al.	+	+	?	?	+	+	?

(+) low risk; (?) some concerns; (−) high risk

### Network meta-analysis

#### Operation time

Data on 666 patients, operated with CS fixation, were pooled from 7 RCTs [31, 32, 38, 41, 44, 47, 52], data on 150 patients, operated with DHS fixation, were pooled from 2 RCTs [41, 56], data on 1031 patients, operated with HA, were pooled from 17 RCTs [25, 27–29, 31, 32, 35, 38, 39, 44, 45, 47–50, 54, 56], and data on 517 patients, operated with THA, were pooled from 12 RCTs [25, 27–29, 35, 39, 45, 48–50, 52, 55]. The operation time of THA was 84.7 min. CS fixation had significantly shorter operation time of 57.7 min. than THA; DHS fixation had significantly shorter operation time of 53.6 min. than THA, HA had significantly shorter operation time of 20.9 min. than THA (CS: MD = − 57.70, 95% CI − 72.78; − 42.62; DHS: MD = − 53.56, 95% CI − 76.17; − 30.95; HA: MD = − 20.90, 95% CI − 30.65; − 11.15; THA: MD = 0.00 reference; Fig. 2). The global consistency assumption of the network meta-analysis model was not met. However, the local consistency assumption was met, suggesting no significant design-by-treatment interaction.

#### Intraoperative blood loss

Data on 543 patients, operated with CS fixation, were pooled from 5 RCTs [31, 32, 38, 44, 52], data on 21 patients, operated with DHS fixation, were pooled from 1 RCT [56], data on 841 patients, operated with HA, were pooled from 12 RCTs [27, 29, 31, 32, 38, 44, 45, 48–50, 54, 56], and data on 383 patients, operated with THA, were pooled from 8 RCTs [27, 29, 45, 48–50, 52, 54]. The intraoperative blood loss of THA was 400 ml. CS fixation had significantly lower intraoperative blood loss of 367 ml than THA; DHS fixation had significantly lower intraoperative blood loss of 320 ml than THA, HA had significantly lower intraoperative blood loss of 120 ml than THA (CS: MD = − 3.67, 95% CI − 4.44; − 2.90; DHS: MD = − 3.20, 95% CI − 4.97; − 1.43; HA: MD = − 1.20, 95% CI − 1.73; − 0.67; THA: MD = 0.00 reference; Fig. 3). The global and local consistency assumption of the network meta-analysis model was not met. A net heat plot showed that the inclusion of the RCT by Tidermark et al. [52] resulted in the greatest heterogeneity. However, the model without the RCT by Tidermark et al. [52] showed comparable estimates to the model including all RCTs.

#### EQ 5D 3–4 months postoperatively

Data on 223 patients, operated with CS fixation, were pooled from 2 RCTs [31, 32], data on 338 patients, operated with HA, were pooled from 4 RCTs [29, 31, 32, 34], and data on 120 patients, operated with THA, were pooled from 2 RCTs [29, 34]. There was no significant difference in EQ 5D 3–4 months postoperatively between CS fixation, HA, and THA (CS: MD = − 0.08, 95% CI − 0.17; 0.00; HA: MD = − 0.01, 95% CI − 0.08; 0.06; THA: MD = 0.00 reference; Fig. 4).

#### EQ 5D 12 months postoperatively

Data on 223 patients, operated with CS fixation, were pooled from 2 RCTs [31, 32], data on 338 patients, operated with HA, were pooled from 4 RCTs [29, 31, 32, 34], and data on 120 patients, operated with THA, were pooled from 2 RCTs [29, 34]. CS fixation had significantly lower EQ 5D 12 months postoperatively of 0.11 points than THA. There was no significant difference in EQ 5D 12 months postoperatively between HA and THA (CS: MD = − 0.11, 95% CI − 0.21; − 0.02; HA: MD = − 0.03, 95% CI − 0.11; 0.04; THA: MD = 0.00 reference; Fig. 5).

#### EQ 5D 2 years postoperatively

Data on 223 patients, operated with CS fixation, were pooled from 2 RCTs [31, 32], data on 338 patients, operated with HA, were pooled from 4 RCTs [29, 31, 32, 34], and data on 120 patients, operated with THA, were pooled from 2 RCTs [29, 34]. CS fixation had significantly lower EQ 5D 2 years postoperatively of 0.20 points than THA. HA had significantly lower EQ 5D 2 years postoperatively of 0.09 points than THA (CS: MD = − 0.20, 95% CI − 0.29; − 0.11; HA: MD = − 0.09, 95% CI − 0.17; − 0.02; THA: MD = 0.00 reference; Fig. 6).

#### Harris Hip Score ≤ 6 months postoperatively

Data on 434 patients, operated with CS fixation, were pooled from 5 RCTs [31, 32, 38, 51, 57], data on 138 patients, operated with DHS fixation, were pooled from 2 RCTs [42, 57], data on 794 patients, operated with HA, were pooled from 11 RCTs [27–29, 31, 32, 34, 38, 39, 42, 51, 54], and data on 425 patients, operated with THA, were pooled from 7 RCTs [27–29, 34, 39, 42, 54]. Two [54, 57] of those RCTs provided data at different time points (HHS ≤ 3 and 6 months postoperatively). CS fixation had significantly lower HHS ≤ 6 months postoperatively of 6.26 points than THA. DHS fixation had significantly lower HHS ≤ 6 months postoperatively of 5.02 points than THA. There was no significant difference in HHS ≤ 6 months postoperatively between HA and THA (CS: MD = − 6.26, 95% CI − 9.74; − 2.79; DHS: MD = − 5.02, 95% CI − 8.78; − 1.26; HA: MD = − 2.00, 95% CI − 4.10; 0.09; THA: MD = 0.00 reference; Fig. 7). The global and local consistency assumption of the network meta-analysis model was not met. A net heat plot showed that the inclusion of the RCT by Watson et al. [57] resulted in the greatest heterogeneity. The model without the RCT by Watson et al. [57] only showed different results for the comparison of DHA and THA, i.e., a non-significant difference between the two treatments (MD = − 1.80, 95% CI − 4.06; 0.47).

#### Harris Hip Score 12 months postoperatively

Data on 405 patients, operated with CS fixation, were pooled from 5 RCTs [31, 32, 38, 51, 57], data on 200 patients, operated with DHS fixation, were pooled from 3 RCTs [31, 42, 57], data on 1098 patients, operated with HA, were pooled from 14 RCTs [27–32, 34, 38, 39, 42, 49, 51, 54, 55], and data on 520 patients, operated with THA, were pooled from 9 RCTs [27–29, 34, 39, 42, 49, 54, 55]. CS fixation had significantly lower HHS 12 months postoperatively of 7.27 points than THA. DHS fixation had significantly lower HHS 12 months postoperatively of 11.56 points than THA. HA had significantly lower HHS 12 months postoperatively of 4.33 points than THA (CS: MD = − 7.27, 95% CI − 11.92; − 2.61; DHS: MD = − 11.56, 95% CI − 16.36; − 6.76; HA: MD = − 4.33, 95% CI − 7.05; − 1.61; THA: MD = 0.00 reference; Fig. 8). The global and local consistency assumption of the network meta-analysis model was not met. A net heat plot showed that the inclusion of the RCTs by Watson et al. [57] and Davison et al. [30] resulted in the greatest heterogeneity. However, the network model without the two RCTs [30, 57] showed comparable estimates to the network model including all RCTs.

#### Harris Hip Score 2 years postoperatively

Data on 405 patients, operated with CS fixation, were pooled from 5 RCTs [31, 32, 38, 51, 57], data on 124 patients, operated with DHS fixation, were pooled from 2 RCTs [30, 57], data on 765 patients, operated with HA were pooled from 10 RCTs [28–32, 34, 38, 40, 50, 51], and data on 205 patients, operated with THA, were pooled from 5 RCTs [28, 29, 34, 40, 50]. CS fixation had significantly lower HHS 2 years postoperatively of 5.50 points than THA. DHS fixation had significantly lower HHS 2 years postoperatively of 8.93 points than THA. HA had significantly lower HHS 2 years postoperatively of 3.65 points than THA (CS: MD = − 5.50, 95% CI − 9.98; − 1.03; DHS: MD = − 8.93, 95% CI − 15.08; − 2.78; HA: MD = − 3.65, 95% CI − 6.74; − 0.57; THA: MD = 0.00 reference; Fig. 9).

#### Harris Hip Score 3–5 years postoperatively

Data on 123 patients, operated with CS fixation, were pooled from 1 RCT [38], data on 355 patients, operated with DHS fixation, were pooled from 2 RCTs [30, 42], data on 1035 patients, operated with HA were pooled from 7 RCTs [28, 30, 34, 38, 42, 54, 55], and data on 343 patients, operated with THA were pooled from 5 RCTs [28, 34, 42, 54, 55]. Two [30, 38] of these RCTs provided data at different time points (HHS 3, 4, and 5 years postoperatively). There was no significant difference in HHS 3–5 years postoperatively between CS fixation and THA. DHS fixation had significantly lower HHS 3–5 years postoperatively of 7.17 points than THA. HA had significantly lower HHS 3–5 years postoperatively of 3.73 points than THA. (CS: MD = − 2.92, 95% CI − 8.76; 2.91; DHS: MD = − 7.17, 95% CI − 11.70; − 2.64; HA: MD = − 3.73, 95% CI − 7.05; − 0.42; THA: MD = 0.00 reference; Fig. 10). The global consistency assumption of the network meta-analysis model was not met. However, the local consistency assumption was met, suggesting no significant design-by-treatment interaction.

#### Hospital stay

Data on 342 patients, operated with CS fixation, were pooled from 5 RCTs [31, 32, 37, 38, 47], data on 169 patients, operated with DHS fixation, were pooled from 2 RCTs [30, 42], data on 941 patients, operated with HA were pooled from 13 RCTs [28, 30–32, 35, 38, 39, 42, 45, 47–49, 55], and data on 454 patients, operated with THA were pooled from 9 RCTs [28, 35, 37, 39, 42, 45, 48, 49, 55]. CS fixation had significantly shorter hospital stay of 2.97 days than THA. There was no significant difference in hospital stay between DHS fixation, HA, and THA (CS: MD = − 2.97, 95% CI − 5.33; − 0.61; DHS: MD = 1.30, 95% CI − 1.64; 4.23; HA: MD = − 0.83, 95% CI − 2.40; 0.75; THA: MD = 0.00 reference; Fig. 11). The global consistency assumption of the network meta-analysis model was not met. However local consistency assumption was met, suggesting no significant design-by-treatment interaction.

#### Reoperation

Data on 493 patients, operated with CS fixation, were pooled from 7 RCTs [31, 32, 36–38, 41, 47], data on 210 patients, operated with DHS fixation, were pooled from 3 RCTs [41, 46, 56], data on 1590 patients, operated with HA, were pooled from 15 RCTs [25, 27–29, 31–33, 35, 38, 39, 46–48, 55, 56], and data on 1305 patients, operated with THA, were pooled from 12 RCTs [25, 27–29, 33, 35–37, 39, 46, 48, 55]. CS fixation had a 9.98 times significantly higher reoperation risk than THA; DHS fixation had a 5.07 times significantly higher reoperation risk than THA; there was no significant difference in reoperation risk between HA and THA (CS: OR = 9.98, 95% CI 4.60; 21.63; DHS: OR = 5.07, 95% CI 2.15; 11.96; HA: OR = 1.60, 95% CI 0.89; 2.89; THA: OR = 1.00 reference; Fig. 12). The analysis of all RCTs together showed that the global consistency assumption of the network meta-analysis model was not met. A net heat plot showed that the inclusion of the RCT by Ravikumar et al. [46] resulted in the greatest heterogeneity. However, the model without the RCT by Ravikumar et al. [46] showed comparable estimates to the model including all RCTs.

#### Mortality

Data on 427 patients, operated with CS fixation, were pooled from 5 RCTs [31, 32, 36, 41, 52], data on 248 patients, operated with DHS fixation, were pooled from 4 RCTs [41, 42, 46, 56], data on 1533 patients, operated with HA, were pooled from 14 RCTs [25, 27, 31–33, 35, 39, 42, 45, 46, 48, 54–56], and data on 1358 patients, operated with THA, were pooled from 13 RCTs [25, 27, 33, 35, 36, 39, 42, 45, 46, 48, 52, 54, 55]. There was no significant difference in mortality risk between CS fixation, DHS fixation, HA, and THA (CS: OR = 1.16, 95% CI 0.82; 1.64; DHS: OR = 0.98, 95% CI 0.63; 1.53; HA: OR = 0.89, 95% CI 0.73; 1.10; THA: OR = 1.00 reference; Fig. 13).

#### Deep vein thrombosis

Data on 413 patients, operated with CS fixation, were pooled from 4 RCTs [32, 36, 37, 44], data on 564 patients, operated with HA, were pooled from 6 RCTs [27, 29, 32, 44, 45, 48], and data on 301 patients, operated with THA, were pooled from 6 RCTs [27, 29, 36, 37, 45, 48]. There was no significant difference in deep vein thrombosis risk between CS fixation, HA, and THA (CS: OR = 1.22, 95% CI 0.26; 5.78; HA: OR = 0.96, 95% CI 0.24; 3.84; THA: OR = 1.00 reference; Fig. 14).

#### Hematoma

Data on 226 patients, operated with CS fixation, were pooled from 1 RCT [44], data on 21 patients, operated with DHS fixation, were pooled from 1 RCT [56], data on 356 patients, operated with HA, were pooled from 4 RCTs [44, 45, 48, 56], and data on 106 patients, operated with THA, were pooled from 2 RCTs [45, 48]. There was no significant difference in hematoma risk between CS fixation, DHS fixation, HA, and THA (CS: OR = 0.14, 95% CI 0.00; 6.15; DHS: OR = 0.45, 95% CI 0.01; 14.10; HA: OR = 0.43, 95% CI 0.06; 3.00; THA: OR = 1.00 reference; Fig. 15).

#### Infection

Data on 618 patients, operated with CS fixation, were pooled from 7 RCTs [31, 32, 36–38, 44, 47], data on 205 patients, operated with DHS fixation, were pooled from 3 RCTs [30, 46, 56], data on 1819 patients, operated with HA, were pooled from 14 RCTs [27, 29–33, 38, 44–49, 56], and data on 1148 patients, operated with THA, were pooled from 9 RCTs [27, 28, 34, 36, 37, 45, 46, 48, 49]. There was no significant difference in infection risk between CS fixation, DHS fixation, HA, and THA (CS: OR = 0.61, 95% CI 0.28; 1.35; DHS: OR = 0.87, 95% CI 0.31; 2.44; HA: OR = 1.01, 95% CI 0.64; 1.58; THA: OR = 1.00 reference; Fig. 16).

#### Intraoperative fracture

Data on 111 patients, operated with CS fixation, were pooled from 1 RCT [31], data on 943 patients, operated with HA, were pooled from 4 RCTs [29, 31, 33, 48], and data on 832 patients, operated with THA, were pooled from 3 RCTs [29, 33, 48]. There was no significant difference in intraoperative fracture risk between CS fixation, HA, and THA (CS: OR = 2.73, 95% CI 0.11; 70.13; HA: OR = 0.93, 95% CI 0.59; 1.47; THA: OR = 1.00 reference; Fig. 17).

#### Failure

Data on 468 patients, operated with CS fixation, were pooled from 6 RCTs [31, 32, 36, 38, 41, 47], data on 212 patients, operated with DHS fixation, were pooled from 3 RCTs [30, 41, 56], data on 1423 patients, operated with HA, were pooled from 9 RCTs [30–33, 38, 47, 48, 55, 56], and data on 937 patients, operated with THA, were pooled from 4 RCTs [33, 36, 48, 55]. CS fixation had a 17.15 times significantly higher failure risk than THA; DHS fixation had a 16.62 times significantly higher failure risk than THA; there was no significant difference in failure risk between HA and THA (CS: OR = 17.15, 95% CI 5.48; 53.70; DHS: OR = 16.62, 95% CI 4.54; 60.82; HA: OR = 1.25, 95% CI 0.49; 3.20; THA: OR = 1.00 reference; Fig. 18).

#### Dislocation

Data on 240 patients, operated with CS fixation, were pooled from 4 RCTs [32, 36, 37, 47], data on 91 patients, operated with DHS fixation, were pooled from 1 RCT [46], data on 1416 patients, operated with HA, were pooled from 13 RCTs [25, 28, 29, 32, 33, 35, 39, 43, 46–48, 50, 55], and data on 1295 patients, operated with THA, were pooled from 13 RCTs [25, 28, 29, 33, 35–37, 39, 43, 46, 48, 50, 55]. CS fixation had a 94% significantly lower dislocation risk than THA; DHS fixation had a 98% significantly lower dislocation risk than THA; HA had a 52% significantly lower dislocation risk than THA (CS: OR = 0.06, 95% CI 0.01; 0.30; DHS: OR = 0.02, 95% CI 0.00; 0.33; HA: OR = 0.48, 95% CI 0.28; 0.81; THA: OR = 1.00 reference; Fig. 19).

#### ANFH

Data on 418 patients, operated with CS fixation, were pooled from 5 RCTs [31, 32, 38, 41, 47], and data on 191 patients, operated with DHS fixation, were pooled from 2 RCTs [30, 41]. There was no significant difference in ANFH risk between DHS and CS (DHS: OR = 1.52, 95% CI 0.54; 4.30; CS: OR = 1.00 reference; Fig. 20).

#### Nonunion

Data on 328 patients, operated with CS fixation, were pooled from 5 RCTs [31, 36–38, 41], and data on 191 patients, operated with DHS fixation, were pooled from 2 RCTs [30, 41]. There was no significant difference in nonunion risk between DHS and CS (DHS: OR = 0.73, 95% CI 0.20; 2.59; CS: OR = 1.00 reference; Fig. 21).

### Further results

The 2006 RCT by Baker et al. [26] with a 3-year follow-up, which is continued by the 2011 RCT by Avery et al. [25] with a longer follow-up of almost 9 years provided data on acetabular cup erosion. Three years after implantation, 21 (51.22%) of 41 HAs and 0 of 40 THAs had experienced acetabular erosion [26]. The RCT by Tol et al. [53] provided data on reoperation and dislocation. There were no events for any of the outcome parameters in either the HA or THA groups. Therefore, the data from these two RCTs [26, 53] were presented descriptively and could not be meta-analyzed.

Table 4 shows a ranking of the outcome parameters examined and the rankings generally follow the results of the mixed-effects models for all outcomes except reoperation and failure, which could be explained by the very large uncertainty around the ORs estimated with the models.

**Table 4:** Ranking of probabilities of operative procedures for being best, second, third best, or worst for all investigated outcomes

	CS	DHS	HA	THA
Operation time	Best: 0.884	2nd: 0.782	3rd: 0.333	Worst: 0
Intraoperative blood loss	Best: 0.897	2nd: 0.766	3rd: 0.337	Worst: 0
EQ-5D 3–4 months	worst: 0.017	-	2nd: 0.692	best: 0.791
EQ-5D 12 months	worst: 0.004	-	2nd: 0.587	best: 0.909
EQ-5D 2 years	Worst: 0	–	2nd: 0.504	Best: 0.996
HHS ≤ 6 months	Worst: 0.088	3rd: 0.259	2nd: 0.671	Best: 0.982
HHS 12 months	3rd: 0.337	Worst: 0.020	2nd: 0.644	Best: 0.999
HHS 2 years	3rd: 0.338	Worst: 0.046	2nd: 0.625	Best: 0.991
HHS 3–5 years	2nd: 0.571	Worst: 0.034	3rd: 0.456	Best: 0.939
Hospital stay	Best: 0.992	Worst: 0.091	2nd: 0.610	3rd: 0.307
Reoperation	Worst: 0.348	Best: 0.652	3rd: 0.360	2nd: 0.640
Mortality	Worst: 0.169	2nd: 0.544	Best: 0.808	3rd: 0.479
Deep vein thrombosis	Worst: 0.382	–	Best: 0.565	2nd: 0.553
Haematoma	Best: 0.764	3rd: 0.490	2nd: 0.525	Worst: 0.221
Infection	Best: 0.846	2nd: 0.507	Worst: 0.323	3rd: 0.325
Intraoperative fracture	–	–	Best: 0.685	Worst: 0.563
Loosening	3rd: 0.489	2nd: 0.511	Worst: 0.447	Best: 0.553
Dislocation	2nd: 0.743	Best: 0.918	3rd: 0.337	Worst: 0.002
ANFH	Best: 0.406	Worst: 0.094	–	–
Nonunion	Worst: 0.101	Best: 0.239	–	–

CS, cannulated screw; DHS, dynamic hip screw; HA, hemiarthroplasty; THA, total hip arthroplasty

The subgroup analysis performed only on displaced femoral neck fractures showed no relevant differences to the network meta-analysis of displaced and non-displaced femoral neck fractures. The forest plots of the subgroup analysis are available in the Additional files 3, 4, 5, 6, 7, 8, 9, 10, 11, 12, 13, 14, 15, 16, 17, 18. There were no RCTs with non-displaced femoral neck fractures for the following outcomes: deep vein thrombosis, hematoma, and dislocation. Therefore, separate subgroup network meta-analyses were not performed. There was only one RCT with non-displaced femoral neck fractures for the intraoperative fracture outcome. Again, separate subgroup network meta-analyses were not performed. The direct comparisons did not show any relevant differences compared with the results of the network meta-analysis. The results of the direct comparisons are shown in the corresponding forest plots (Figs. 2, 3, 4, 5, 6, 7, 8, 9, 10, 11, 12, 13, 14, 15, 16, 17, 18, 19, 20, 21). In addition, a sensitivity analysis to assess the effect of our imputation procedure also showed no relevant differences.

## Discussion

In our study, using a high-quality network meta-analysis, we attempted to rank 4 different operative procedures in patients with femoral neck fractures, distinguishing between displaced and non-displaced fractures. Our overall findings showed that CS fixation was best in terms of operation time and intraoperative blood loss. For quality of life (EQ-5D) and functional outcome (HHS), THA ranked first and HA ranked second. In contrast, CS fixation had the highest reoperation risk, followed by DHS fixation. THA and HA had a low reoperation risk. There was no significant difference in mortality between the 4 operative procedures. The distinction between displaced and non-displaced fractures showed no relevant differences in our network meta-analysis. However, an analysis specifically considering the Pauwels or Garden classification was not possible. On the basis of these findings, we recommend that prosthetic procedures should be preferred in patients with femoral neck fractures. Accordingly, osteosynthesis should only be considered on a case-by-case basis when known factors such as patient age, fracture morphology, patient orientation and compliance, and the expectation of postoperative functional recovery make the preservation of the femoral head possible and absolutely necessary.

To date, numerous studies and meta-analyses have been conducted on this important but still controversial topic. It is striking that the conclusions and recommendations are not consistent. In summary, there is a fair degree of consensus that HA is associated with better overall patient outcomes compared with internal fixation in patients with femoral neck fractures [6, 8, 15]. However, the comparisons between HA and THA do not allow to draw uniform conclusions. Many meta-analyses consider THA to be superior to HA in patients with femoral neck fractures [11–13]. On the other hand, many other meta-analyses find no relevant overall difference in outcome between HA and THA in patients with femoral neck fractures [10, 14, 65–67].

Our study showed that CS fixation had the shortest operation time, followed by DHS fixation, HA, and finally THA. The mean operation time of the 4 operative procedures was: 26.6 min. for CS fixation, 38.8 min. for DHS fixation, 63.2 min. for HA, and 84.7 min. for THA. This ranking by operation time reflects the complexity of each operative procedure. The importance of the outcome parameter is controversial. The operation time itself may not be a decisive outcome parameter, but on the other hand, it is known that longer operation times are associated with higher infection rates and tissue trauma [64]. In 89,802 cases of THA, Surace et al. suggested an optimal operation time of approximately 80 min with a lower risk of perioperative complications [64]. Our results are consistent with the literature. Four recent meta-analyses found a significantly shorter operation time (mean: 12.28–20.04 min.) for HA compared with THA [14, 65–67]. Two other meta-analyses found a significantly shorter operation time (MD: 2.50–36.22 min.) for internal fixation compared with HA [6, 8]. The subgroup analysis showed no relevant differences between displaced and non-displaced femoral neck fractures for the outcome parameter operation time. This may be due to the fact that the reduction of the fracture is usually performed on the traction table shortly before the start of the internal fixation.

The outcome parameter of intraoperative blood loss showed the same ranking of the operative procedures as for operation time, with CS fixation being the best and THA the worst. The mean intraoperative blood loss of the 4 operative procedures was: 30 ml for CS fixation, 200 ml for DHS fixation, 260 ml for HA, and 400 ml for THA. A correlation between the operation time and intraoperative blood loss seems quite likely, as a recent study has convincingly shown [68]. Our results are in line with the literature. Three recent meta-analyses found a significantly lower blood loss (MD: 45.63–69.10 ml) for HA compared with THA [65–67]. Another meta-analysis found a significantly less blood loss (MD: 165.84 ml) with CS fixation compared with HA [6]. Subgroup analysis showed no relevant differences in intraoperative blood loss between displaced and non-displaced femoral neck fractures. Our efforts to maintain stable hemoglobin levels begin intraoperatively and should be continued postoperatively, as a recent study has shown [69]. In the management of blood loss, orthogeriatric care may be of great benefit in the elderly population studied [69].

Our study showed that THA had the best EQ-5D score, followed by HA. CS fixation had the worst EQ-5D score. This ranking was repeated in all three intervals examined (EQ-5D 3–4 months, 12 months, and 2 years postoperatively) at which EQ-5D was recorded. None of the RCTs reported EQ-5D scores for DHS fixation. The mean EQ-5D 3–4 months postoperatively of the 3 operative procedures was: 0.5 points for CS fixation, 0.6 points for HA, 0.7 points for THA. The mean EQ-5D 12 months and 2 years postoperatively of the 3 operative procedures were: 0.6 points for CS fixation, 0.6 points for HA, 0.7 points for THA. The EQ-5D is a generic measuring instrument that uses a standardized, preference-based method to assess health status [18]. In a questionnaire, developed by the EuroQol Group, respondents rate their health on scale from 0 (very poor) to 1 (best possible). Our results are in line with the literature. Two recent meta-analyses found a significantly higher EQ-5D score (MD: 0.13 points) for THA compared with HA [10, 14]. Another meta-analysis found no significant difference in EQ-5D between THA and HA up to 1 year after surgery [65]. Our subgroup analysis showed no relevant differences in EQ-5D between displaced and non-displaced femoral neck fractures.

The HHS was developed to assess hip function after surgery [19]. The score ranges from 0 (very poor) to 100 (best possible) points. It accumulates points from the assessment of four aspects: pain, function, degree of deformity, and range of motion of the hip. Hip function was assessed at regular intervals after surgery (HHS ≤ 6 months, 12 months, 2 years, and 3–5 years postoperatively), providing information on short-, middle-, and long-term functional outcomes of the hip after surgery. Notably, THA had the best place in the ranking at every time point measured. HA was ranked second best at almost every time point measured except for HHS 3–5 years postoperatively when CS fixation was ranked second best. In all other cases, the worst and third place was taken by CS fixation and DHS fixation. The mean HHS ≤ 6 months postoperatively of the 4 operative procedures were: 64.5 points for CS fixation, 67.0 points for DHS fixation, 72.5 points for HA, and 78.4 points for THA. The mean HHS 12 months postoperatively of the 4 operative procedures were: 69.8 points for CS fixation, 71.1 points for DHS fixation, 75.2 points for HA, 81.7 points for THA. The mean HHS 2 years postoperatively of the 4 operative procedures was: 71.3 points for CS fixation, 71.8 for DHS fixation, 74.4 points for HA, 80.2 points for THA. The mean HHS 3–5 years postoperatively of the 4 operative procedures was: 80.9 points for CS fixation, 71.1 for DHS fixation, 75.1 points for HA, 81.0 points for THA. The literature shows similar results. Three recent meta-analyses found a significantly higher HHS (MD: 5.05–6.03 points) for THA compared with HA [10, 11, 65]. Another meta-analysis found no significant difference in HHS between HA and THA [67]. In contrast to our results, another meta-analysis showed no significant difference in HHS 1 and 2 years postoperatively between internal fixation and HA [8]. This meta-analysis was limited to patients with non-displaced femoral neck fractures and included data from only 3 studies, one of which was not an RCT [8]. Furthermore, this meta-analysis did not differentiate between CS fixation and DHS fixation in the internal fixation group [8]. Another meta-analysis of non-displaced femoral neck fractures showed a significantly higher HHS 6 months postoperatively for HA compared with CS fixation (MD: 5.05 points) [6]. The same study found no significant difference in HHS 1 and 2 years postoperatively between CS fixation and HA [6]. The importance of the outcome parameter HHS for assessing the functional outcome of hip operations is undisputed. A recent study found that 1 year after hip fracture in elderly patients, significant loss of muscle mass was common, with impaired functional recovery [70]. One treatment approach in this context may be targeted preservation of muscle mass to improve the prognosis in these patients [70]. However, it should be noted that the minimal clinically important difference (MCID) for HHS has been reported in the literature to be no less than 7.9 points [71–74]. In our study, a significantly higher HHS difference than the MCID was found in only two cases. DHS had significantly lower HHS 12 months postoperatively of 11.56 points compared with THA, and significantly lower HHS 2 years postoperatively of 8.93 points compared with THA.

Our study showed that CS fixation had the shortest hospital stay with 8.4 days. HA was the second best, followed by THA, and finally, DHS fixation, although the differences between these three operative procedures were not significant. A recent meta-analysis found no significant difference in hospital stay between THA and HA [14]. Two other meta-analyses showed a significantly longer hospital stay (MD: 0.47–2.36 days) for HA than for THA [65, 66]. Another meta-analysis, including only non-displaced femoral neck fractures, found a significantly shorter hospital stay (MD: 0.80 days) for internal fixation compared with HA [8]. Another meta-analysis, including only non-displaced femoral neck fractures, found a significantly shorter hospital stay (MD: 3.32 days) for CS fixation compared with HA [6].

Our study showed that CS fixation had the highest reoperation risk with 39.15%, followed by DHS with 27.62%, followed by HA with 9.11% and THA with 6.36%. CS fixation had the highest failure risk with 21.79%, followed by DHS fixation with 15.09%, followed by HA with 1.26% and THA with 0.85%. The difference between HA and THA was not significant. HA had a significantly lower dislocation risk than THA, with rates of 3.11% for HA and 6.33% for THA. The following outcome parameters did not show relevant differences: mortality, deep vein thrombosis, hematoma, infection, intraoperative fracture, ANFH, and nonunion. The subgroup analysis showed no relevant differences between displaced and non-displaced femoral neck fractures for the outcome parameters reoperation, mortality, infection, intraoperative fracture, failure, ANFH, and nonunion. In general, our results were consistent with the literature. We compared our findings with 5 meta-analyses that examined the differences between HA and THA in patients with displaced femoral neck fractures [10–14]. A meta-analysis showed no significant difference in mortality risk between HA (rate: 15.13%) and THA (rate: 13.48%) [10]. The authors Burgers et al. found a 2.53 times significantly higher dislocation risk for THA (rate: 8.94%) compared with HA (rate: 3.40%) [10]. Another meta-analysis showed a 62% significantly lower dislocation risk for HA (rate: 2.70%) compared with THA (rate: 8.12%) [11]. The authors Lewis et al. found no significant difference in infection risk between HA and THA [11]. They also found a 1.54 times significantly higher reoperation risk for HA (rate: 8.76%) compared with THA (rate: 5.72%) [11]. Another meta-analysis found no significant difference in mortality risk between HA (rate: 34.67%) and THA (rate: 30.49%) [12]. The authors Liao et al. found a 60% significantly lower reoperation risk for THA (rate: 5.82%) compared with HA (rate: 14.33%) [12].They also found a 2.02 times significantly higher dislocation risk for THA (rate: 10.67%) compared with HA (rate: 5.18%) [12]. They found no significant difference in infection risk between HA and THA [12]. Another meta-analysis showed a 25% significantly lower mortality risk for THA (rate: 12.22%) compared with HA (rate: 15.37%) [13]. The authors Peng et al. found a 54% significantly lower dislocation risk for THA (rate: 4.15%) compared with HA (rate: 9.22%) [13], which is contradictory to the findings in the specialist literature. Furthermore, they found no significant difference in infection, reoperation, and thromboembolic risk between THA and HA [13]. Another meta-analysis showed a 3.31 times significantly higher reoperation risk for HA (rate: 12.28%) compared with THA (rate: 3.92%) [14]. The authors Wang et al. found no significant difference in infection and dislocation risk [14].

We also compared our findings with 3 meta-analyses that examined the differences in HA and THA in patients with displaced and non-displaced femoral neck fractures [65–67]. One meta-analysis found no significant difference in mortality risk between HA and THA [66]. Another meta-analysis comparing dual-mobility THA with HA showed a 3.60 times significantly higher dislocation risk for HA [67]. The authors Ma et al. found a 2.06 times significantly higher reoperation risk for HA compared with THA [67]. Another meta-analysis found no significant differences in thromboembolic, infection, revision, intraoperative fracture, failure, and mortality (1 year postoperatively) risks between HA and THA [65]. The authors Tang et al. found a 1.90 times significantly higher dislocation risk for THA compared with HA [65]. In general, interdisciplinary orthogeriatric care can help us reduce complication rates and mortality [75]. Important perioperative aspects include pain and fluid management, early mobilization, and delirium prevention [75].

A meta-analysis comparing CS fixation with DHS fixation in patients with displaced and non-displaced femoral neck fractures showed a 1.44 times significantly higher reoperation risk for CS fixation (rate: 33.02%) compared with DHS fixation (rate: 21.77%), and a 2.28 times significantly higher failure risk for CS fixation (rate: 13.04%) compared with THA (rate: 5.49%) [76]. Another meta-analysis showed a 4.49 times significantly higher reoperation risk for internal fixation (CS fixation and DHS fixation) compared with HA [8]. The authors Ma et al. found no significant difference in mortality risk (1 year postoperatively) between internal fixation and HA [8]. Another meta-analysis showed a 4.88 times significantly higher reoperation risk for CS fixation compared with HA [6]. The authors Xu et al. found no significant difference in mortality risk between CS fixation and HA [6].

Despite our results that are in line with the literature, we identified several limitations: (1) There was considerable heterogeneity between individual studies for some outcome parameters, which could affect the final results. (2) In some cases, the quality assessment of the studies produced questionable results. (3) Operative skill of the surgeon, intraoperative warming, injection of local anaesthetics and tranexamic acid, use of bone cement, or type of implant could be considered as confounding factors, which may affect the results to some extent. (4) The number of RCTs reporting patients with non-displaced femoral neck fractures was low so some outcome parameters could not be considered. (5) The distinction between closed and open reduction was not considered in the CS fixation and DHS fixation groups.

## Conclusions

In our cohort of patient with displaced and non-displaced femoral neck fractures, the more important outcome parameters such as HHS, EQ-5D, and reoperation risk showed an advantage of THA and HA compared with CS fixation and DHS fixation. Based on these findings, we recommend that hip arthroplasty should be preferred and internal fixation of femoral neck fractures should only be considered in individual cases.

### Supplementary Information

**Additional file 1:** Prisma checklist.

**Additional file 2:** Raw data extraction sheet.

**Additional file 3:** Forest plot of operation time (displaced femoral neck fractures only). CS, cannulated screw; DHS, dynamic hip screw; HA, hemiarthroplasty; THA, total hip arthroplasty; SD, standard deviation; MD, mean difference; CI, confidence interval.

**Additional file 4:** Forest plot of intraoperative blood loss (displaced femoral neck fractures only). Results are shown for a unit of 100 ml. CS, cannulated screw; DHS, dynamic hip screw; HA, hemiarthroplasty; THA, total hip arthroplasty; SD, standard deviation; MD, mean difference; CI, confidence interval.

**Additional file 5:** Forest plot of EQ 5D 3-4 months postoperatively (displaced femoral neck fractures only). CS, cannulated screw; HA, hemiarthroplasty; THA, total hip arthroplasty; SD, standard deviation; MD, mean difference; CI, confidence interval.

**Additional file 6:** Forest plot of EQ 5D 12 months postoperatively (displaced femoral neck fractures only). CS, cannulated screw; HA, hemiarthroplasty; THA, total hip arthroplasty; SD, standard deviation; MD, mean difference; CI, confidence interval.

**Additional file 7:** Forest plot of EQ 5D 2 years postoperatively (displaced femoral neck fractures only). CS, cannulated screw; HA, hemiarthroplasty; THA, total hip arthroplasty; SD, standard deviation; MD, mean difference; CI, confidence interval.

**Additional file 8:** Forest plot of Harris Hip Score ≤ 6 months postoperatively (displaced femoral neck fractures only). CS, cannulated screw; DHS, dynamic hip screw; HA, hemiarthroplasty; THA, total hip arthroplasty; SD, standard deviation; MD, mean difference; CI, confidence interval.

**Additional file 9:** Forest plot of Harris Hip Score 12 months postoperatively (displaced femoral neck fractures only). CS, cannulated screw; DHS, dynamic hip screw; HA, hemiarthroplasty; THA, total hip arthroplasty; SD, standard deviation; MD, mean difference; CI, confidence interval.

**Additional file 10:** Forest plot of Harris Hip Score 2 years postoperatively (displaced femoral neck fractures only). CS, cannulated screw; DHS, dynamic hip screw; HA, hemiarthroplasty; THA, total hip arthroplasty; SD, standard deviation; MD, mean difference; CI, confidence interval.

**Additional file 11:** Forest plot of Harris Hip Score 3-5 years postoperatively (displaced femoral neck fractures only). CS, cannulated screw; DHS, dynamic hip screw; HA, hemiarthroplasty; THA, total hip arthroplasty; SD, standard deviation; MD, mean difference; CI, confidence interval.

**Additional file 12:** Forest plot of hospital stay (displaced femoral neck fractures only). CS, cannulated screw; DHS, dynamic hip screw; HA, hemiarthroplasty; THA, total hip arthroplasty; SD, standard deviation; MD, mean difference; CI, confidence interval.

**Additional file 13:** Forest plot of reoperation (displaced femoral neck fractures only). CS, cannulated screw; DHS, dynamic hip screw; HA, hemiarthroplasty; THA, total hip arthroplasty; OR, odds ratio; CI, confidence interval.

**Additional file 14:** Forest plot of mortality (displaced femoral neck fractures only). CS, cannulated screw; DHS, dynamic hip screw; HA, hemiarthroplasty; THA, total hip arthroplasty; OR, odds ratio; CI, confidence interval.

**Additional file 15:** Forest plot of infection (displaced femoral neck fractures only). CS, cannulated screw; DHS, dynamic hip screw; HA, hemiarthroplasty; THA, total hip arthroplasty; OR, odds ratio; CI, confidence interval.

**Additional file 16:** Forest plot of failure (displaced femoral neck fractures only). CS, cannulated screw; DHS, dynamic hip screw; HA, hemiarthroplasty; THA, total hip arthroplasty; OR, odds ratio; CI, confidence interval.

**Additional file 17:** Forest plot of ANFH (displaced femoral neck fractures only). CS, cannulated screw; DHS, dynamic hip screw; HA, hemiarthroplasty; OR, odds ratio; CI, confidence interval.

**Additional file 18:** Forest plot of nonunion (displaced femoral neck fractures only). CS, cannulated screw; DHS, dynamic hip screw; HA, hemiarthroplasty; THA, total hip arthroplasty; OR, odds ratio; CI, confidence interval.

## Data Availability

Raw data extraction sheet is available in the Additional file 1.

## Abbreviations

ANFH: Avascular necrosis of the femoral head
CS: Cannulated screw(s)
DHS: Dynamic hip screw
EQ-5D: Not an abbreviation
HA: Hemiarthroplasty
HHS: Harris Hip Score
ITT: Intention to treat
MCID: Minimal clinically important difference
RCT: Randomized controlled trial
THA: Total hip arthroplasty
SUCRA: Surface under the cumulative ranking curve
