# Supplementary figures and images for: Cannulated screws versus dynamic hip screw versus hemiarthroplasty versus total hip arthroplasty in patients with displaced and non-displaced femoral neck fractures: a systematic review and frequentist network meta-analysis of 5703 patients
Source: J Orthop Surg Res. 2023 Aug 26;18:625. doi: 10.1186/s13018-023-04114-8 (PMC10464356; doi:10.1186/s13018-023-04114-8)

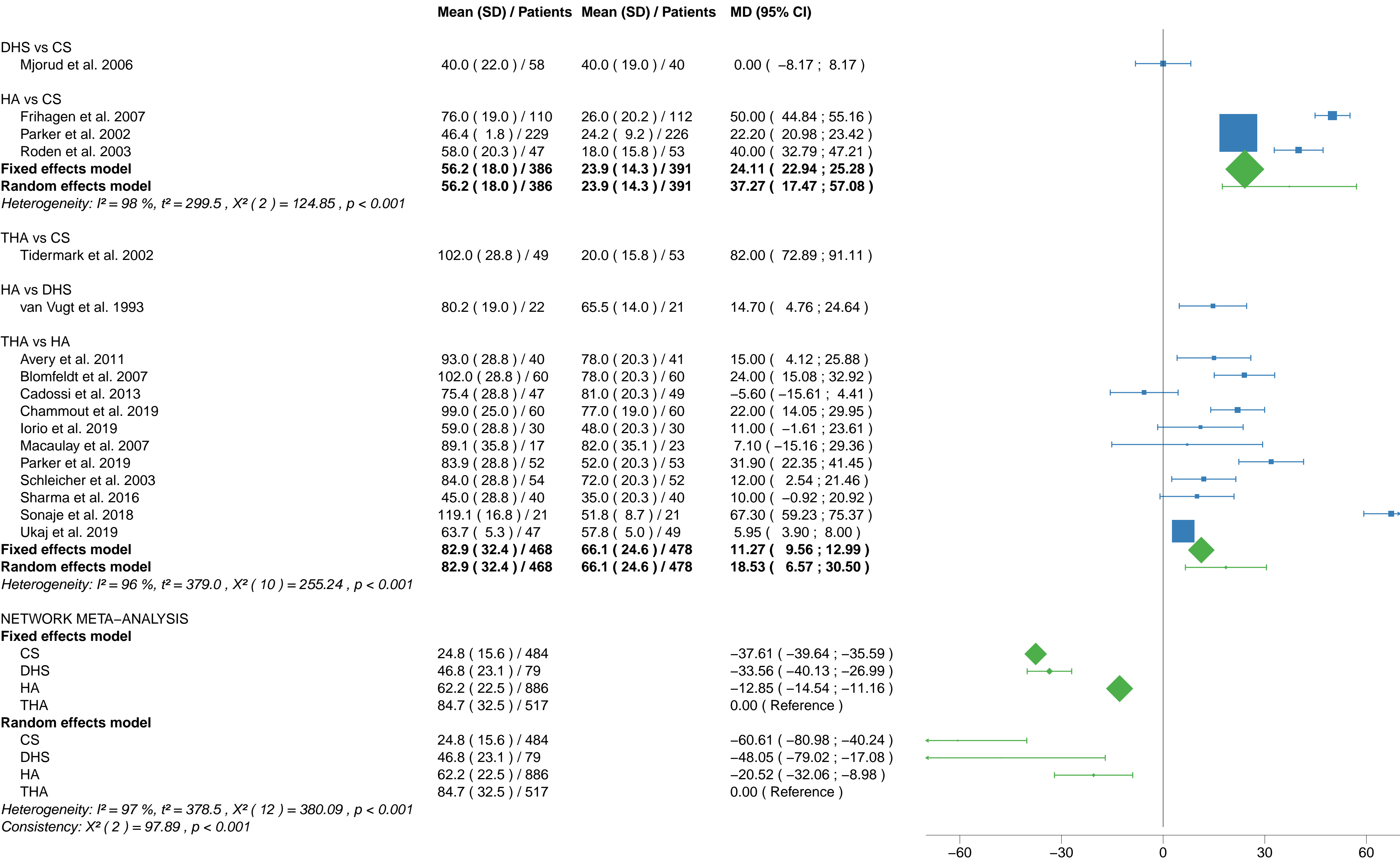

Supplement: Supplementary file 3 — Additional file 3: Forest plot of operation time (displaced femoral neck fractures only). CS, cannulated screw; DHS, dynamic hip screw; HA, hemiarthroplasty; THA, total hip arthroplasty; SD, standard deviation; MD, mean difference; CI, confidence interval. [file 13018_2023_4114_MOESM3_ESM.pdf]

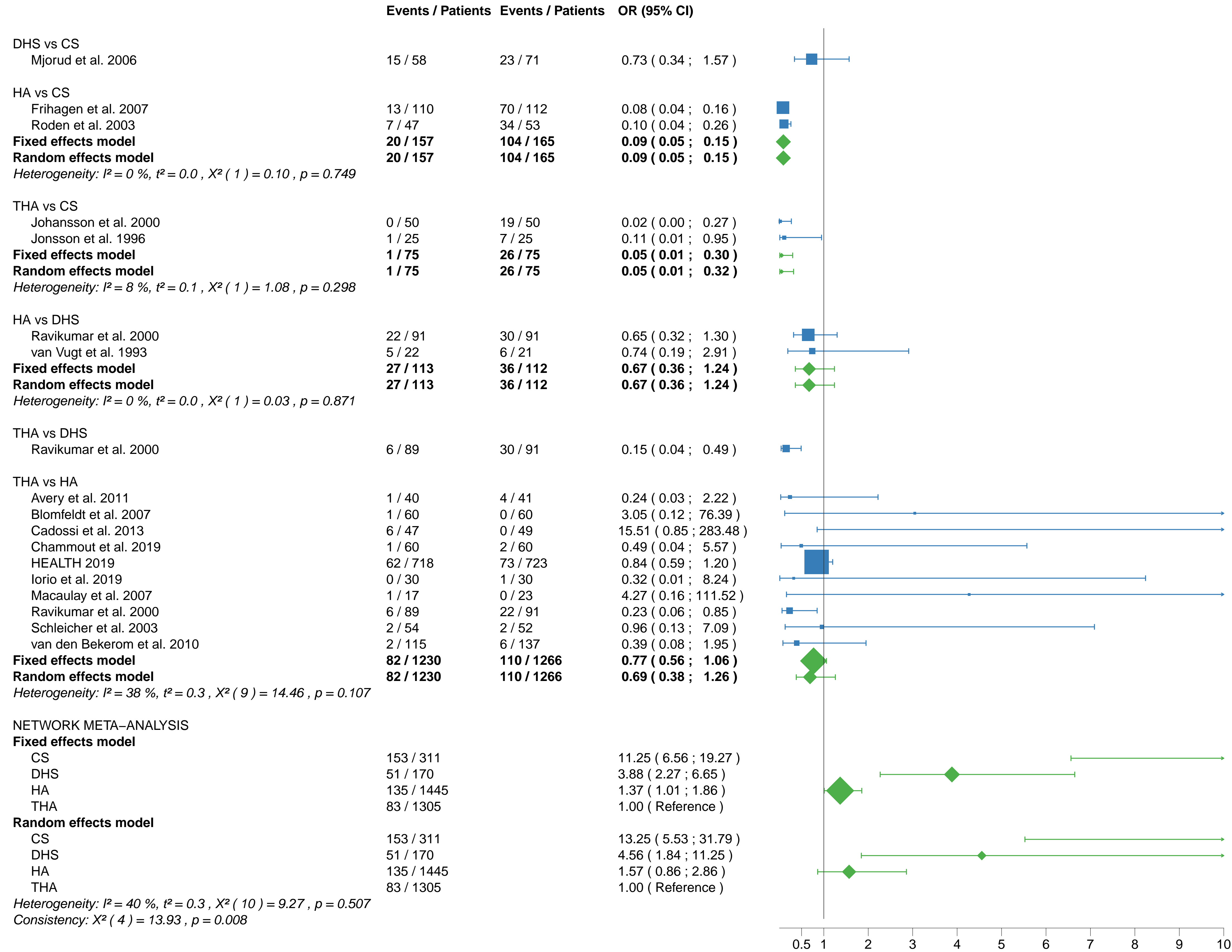

Supplement: Supplementary file 13 — Additional file 13: Forest plot of reoperation (displaced femoral neck fractures only). CS, cannulated screw; DHS, dynamic hip screw; HA, hemiarthroplasty; THA, total hip arthroplasty; OR, odds ratio; CI, confidence interval. [file 13018_2023_4114_MOESM13_ESM.pdf]

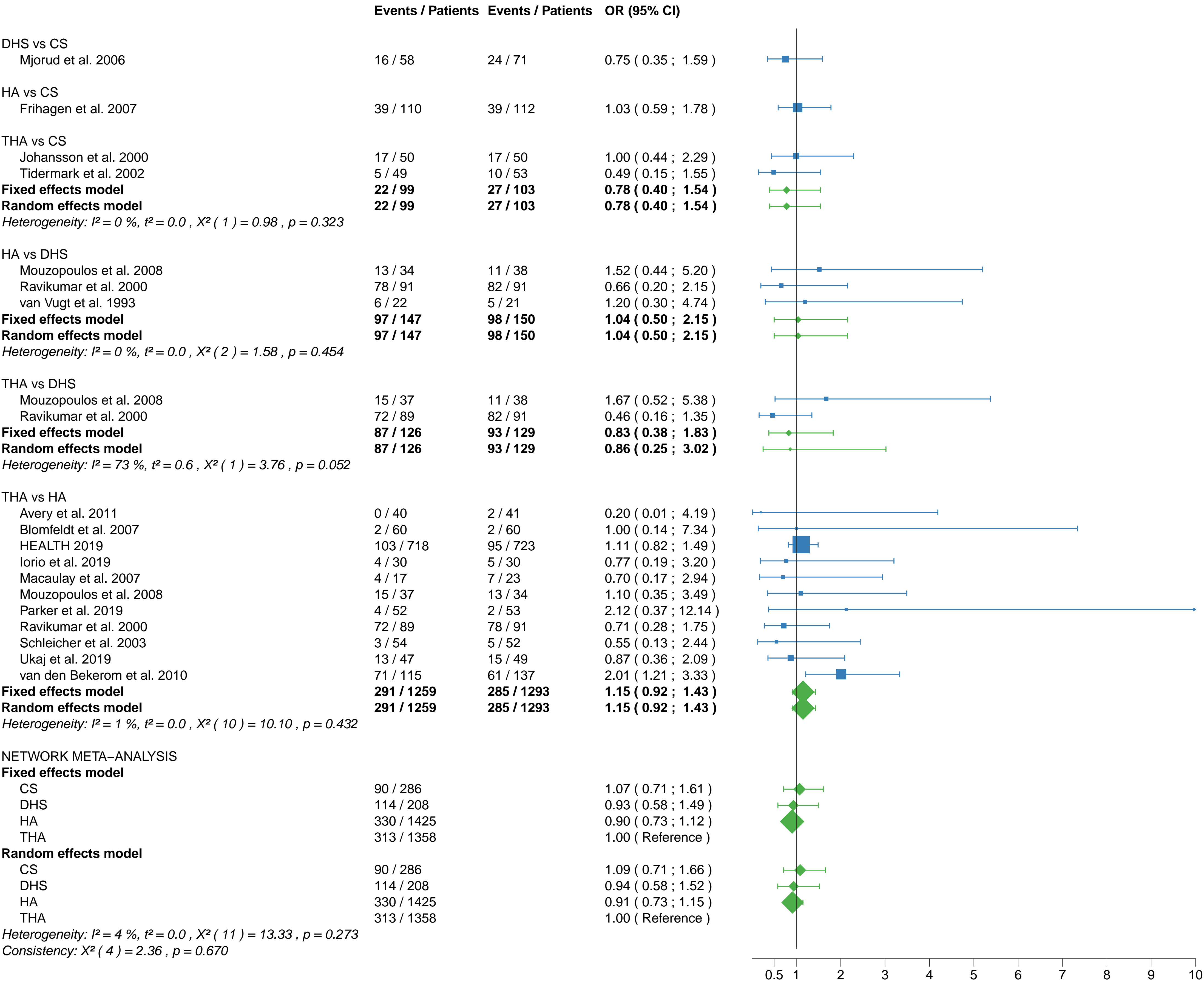

Supplement: Supplementary file 14 — Additional file 14: Forest plot of mortality (displaced femoral neck fractures only). CS, cannulated screw; DHS, dynamic hip screw; HA, hemiarthroplasty; THA, total hip arthroplasty; OR, odds ratio; CI, confidence interval. [file 13018_2023_4114_MOESM14_ESM.pdf]

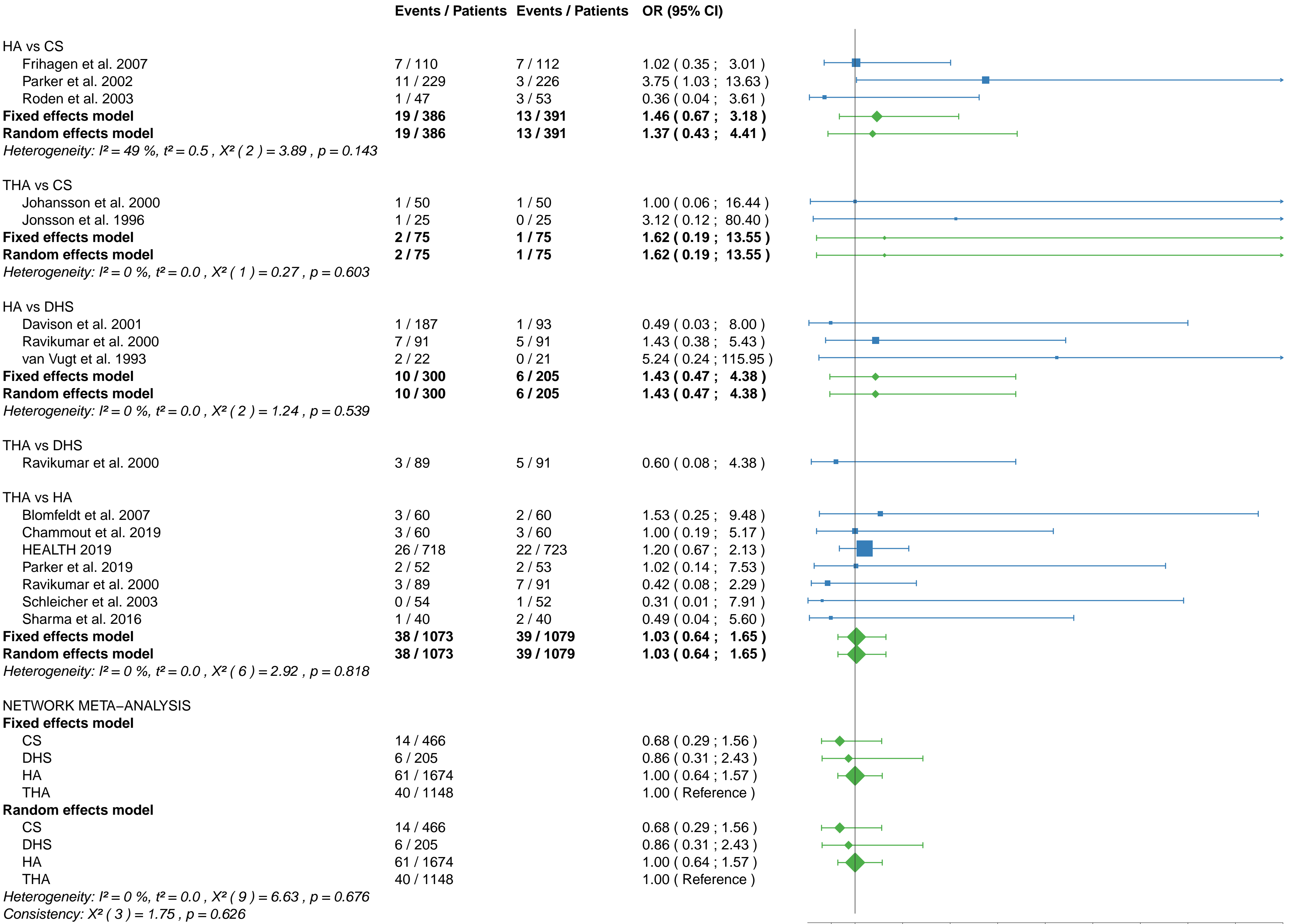

Supplement: Supplementary file 15 — Additional file 15: Forest plot of infection (displaced femoral neck fractures only). CS, cannulated screw; DHS, dynamic hip screw; HA, hemiarthroplasty; THA, total hip arthroplasty; OR, odds ratio; CI, confidence interval. [file 13018_2023_4114_MOESM15_ESM.pdf]

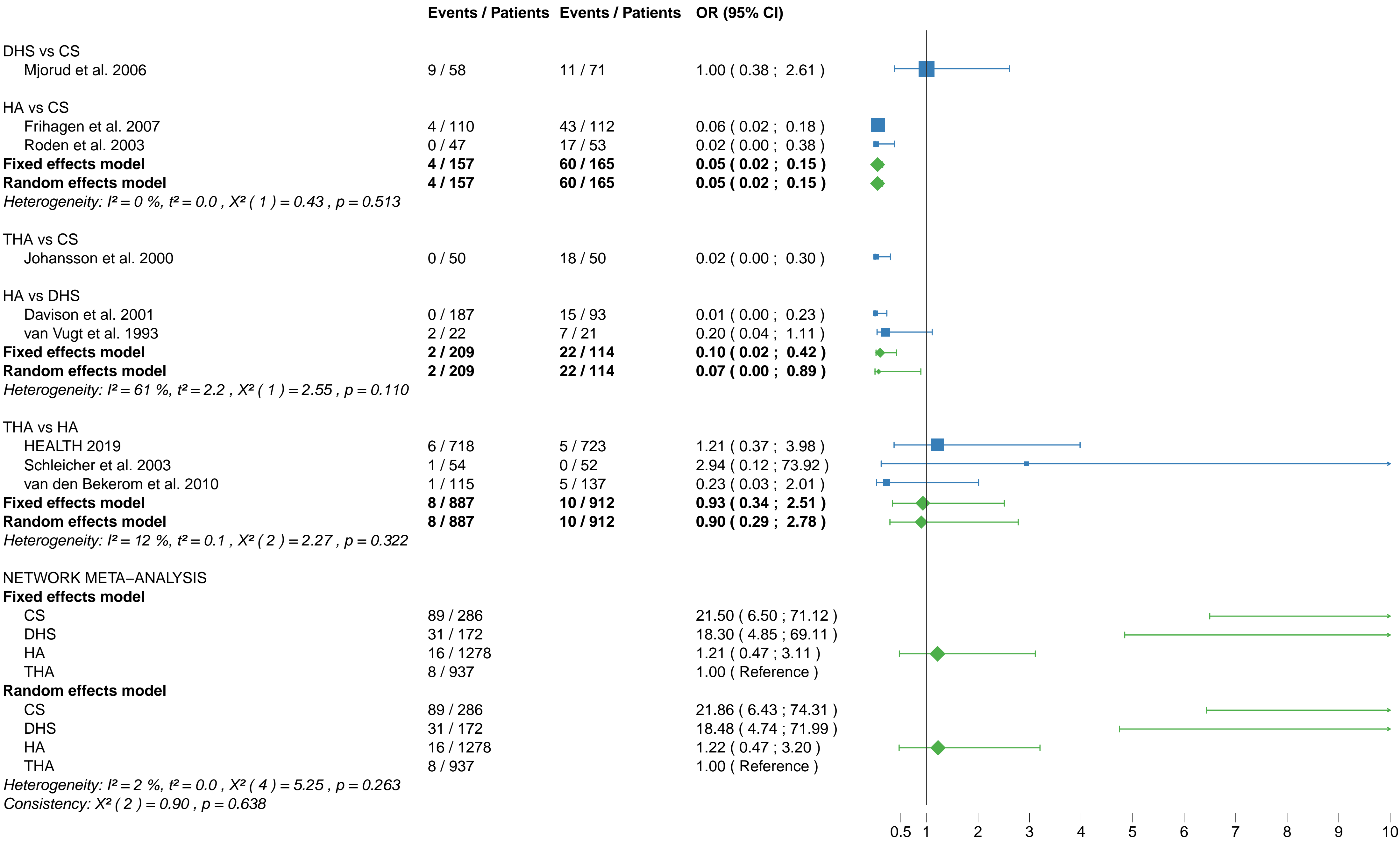

Supplement: Supplementary file 16 — Additional file 16: Forest plot of failure (displaced femoral neck fractures only). CS, cannulated screw; DHS, dynamic hip screw; HA, hemiarthroplasty; THA, total hip arthroplasty; OR, odds ratio; CI, confidence interval. [file 13018_2023_4114_MOESM16_ESM.pdf]

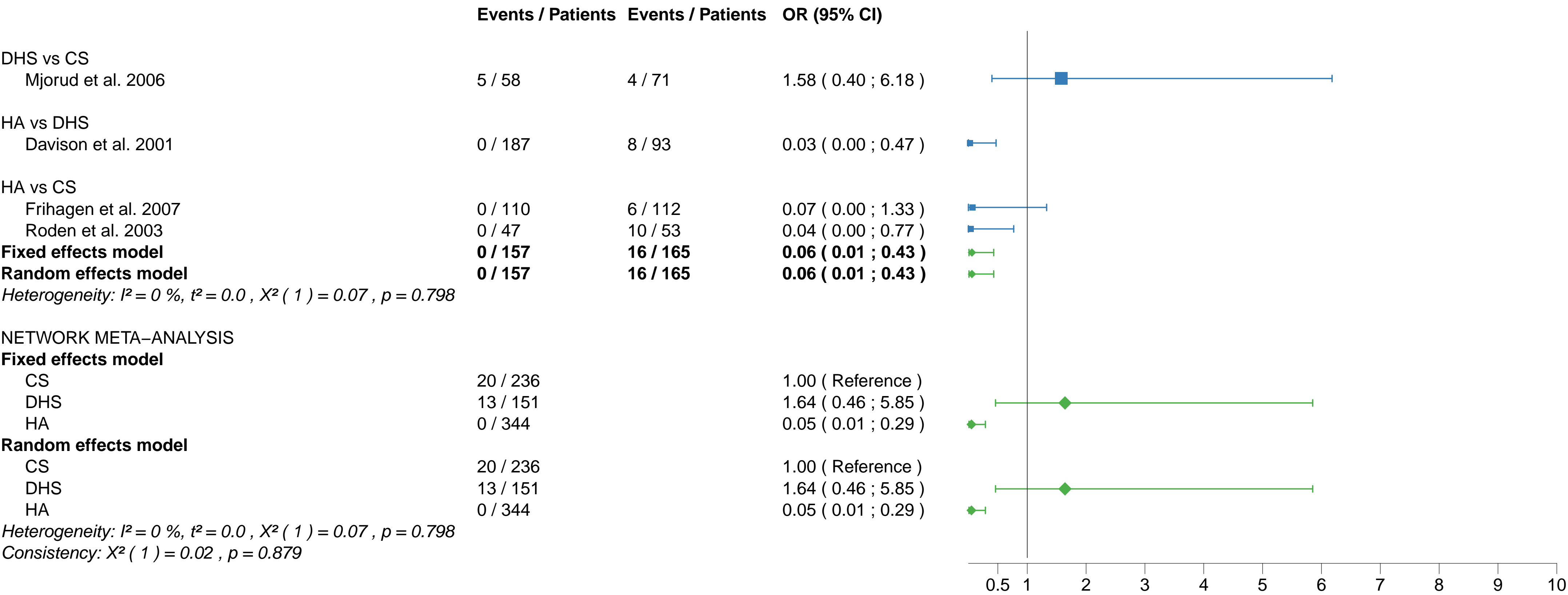

Supplement: Supplementary file 17 — Additional file 17: Forest plot of ANFH (displaced femoral neck fractures only). CS, cannulated screw; DHS, dynamic hip screw; HA, hemiarthroplasty; OR, odds ratio; CI, confidence interval. [file 13018_2023_4114_MOESM17_ESM.pdf]

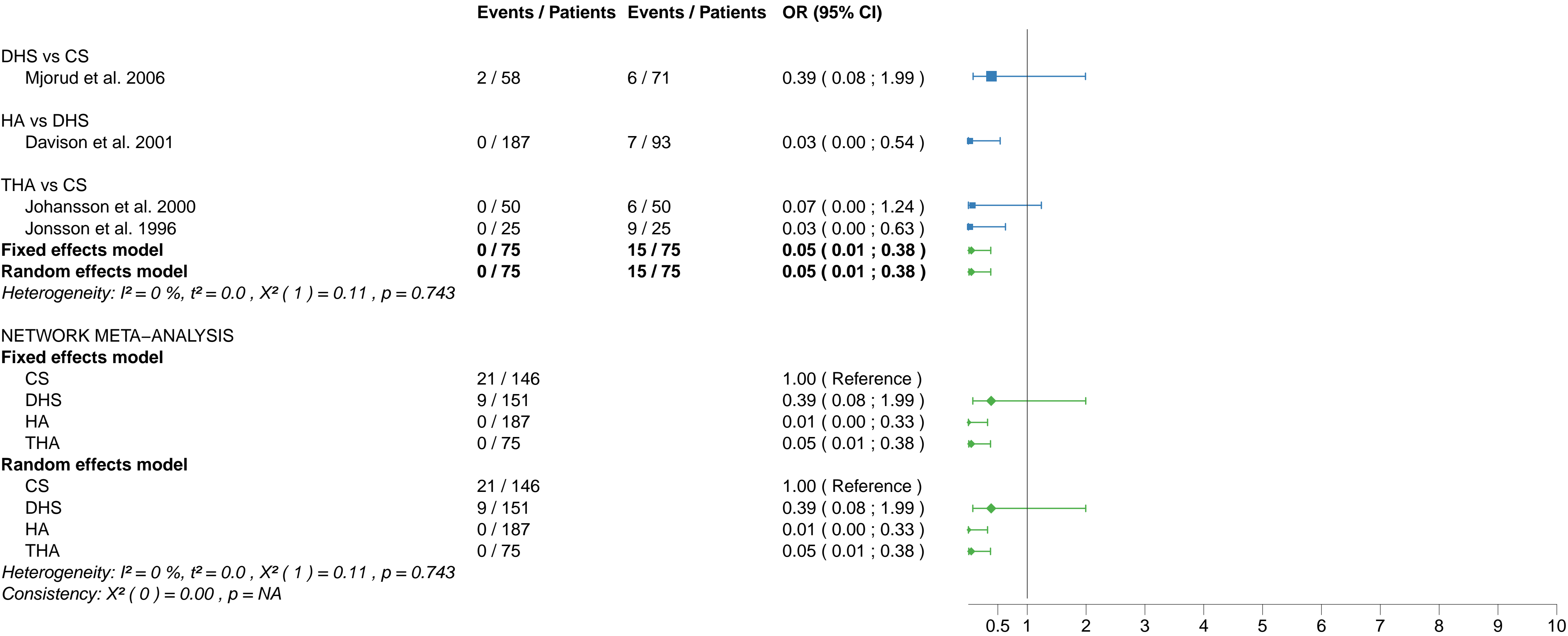

Supplement: Supplementary file 18 — Additional file 18: Forest plot of nonunion (displaced femoral neck fractures only). CS, cannulated screw; DHS, dynamic hip screw; HA, hemiarthroplasty; THA, total hip arthroplasty; OR, odds ratio; CI, confidence interval. [file 13018_2023_4114_MOESM18_ESM.pdf]
