# Supplementary material for: Cannulated screws versus dynamic hip screw versus hemiarthroplasty versus total hip arthroplasty in patients with displaced and non-displaced femoral neck fractures: a systematic review and frequentist network meta-analysis of 5703 patients
Source: J Orthop Surg Res. 2023 Aug 26;18:625. doi: 10.1186/s13018-023-04114-8 (PMC10464356; doi:10.1186/s13018-023-04114-8)

	Mean (SD) / Patients	Mean (SD) / Patients	MD (95% CI)
HA vs CS			
Frihagen et al. 2007	3.5 (2.0) / 110	0.3 (0.9) / 112	3.13 (2.72 ; 3.54)
Parker et al. 2002	1.8 (1.1) / 229	0.3 (0.2) / 226	1.49 (1.34 ; 1.64)
**Fixed effects model**	**2.3 (1.7) / 339**	**0.3 (0.5) / 338**	**1.68 (1.54 ; 1.82)**
**Random effects model**	**2.3 (1.7) / 339**	**0.3 (0.5) / 338**	**2.30 (0.69 ; 3.91)**
Heterogeneity: $I^2 = 98\%$, $t^2 = 1.3$, $X^2(1) = 53.53$, $p < 0.001$			
THA vs CS			
Tidermark et al. 2002	5.5 (1.6) / 49	0.2 (0.5) / 53	5.30 (4.83 ; 5.77)
HA vs DHS			
van Vugt et al. 1993	4.0 (1.6) / 22	2.0 (1.6) / 21	2.00 (1.04 ; 2.96)
THA vs HA			
Blomfeldt et al. 2007	4.6 (1.6) / 60	3.2 (1.6) / 60	1.40 (0.83 ; 1.97)
Chammout et al. 2019	3.5 (2.0) / 60	3.2 (2.2) / 60	0.31 (−0.44 ; 1.06)
Parker et al. 2019	3.4 (1.6) / 52	2.4 (1.6) / 53	0.91 (0.30 ; 1.52)
Schleicher et al. 2003	6.0 (1.6) / 54	4.0 (1.6) / 52	2.00 (1.39 ; 2.61)
Sharma et al. 2016	3.0 (1.6) / 40	2.0 (1.6) / 40	1.00 (0.30 ; 1.70)
Sonaje et al. 2018	3.4 (0.2) / 21	2.4 (0.2) / 21	0.99 (0.85 ; 1.12)
Ukaj et al. 2019	1.8 (0.2) / 47	1.6 (0.3) / 49	0.19 (0.10 ; 0.28)
**Fixed effects model**	**3.8 (2.0) / 334**	**2.8 (1.7) / 335**	**0.49 (0.42 ; 0.57)**
**Random effects model**	**3.8 (2.0) / 334**	**2.8 (1.7) / 335**	**0.95 (0.47 ; 1.43)**
Heterogeneity: $I^2 = 95\%$, $t^2 = 0.3$, $X^2(6) = 131.56$, $p < 0.001$			
NETWORK META−ANALYSIS			
**Fixed effects model**			
CS	0.3 (0.5) / 391		−2.50 (−2.65 ; −2.35)
DHS	2.0 (1.6) / 21		−2.56 (−3.52 ; −1.60)
HA	2.6 (1.7) / 696		−0.56 (−0.64 ; −0.49)
THA	4.0 (2.0) / 383		0.00 (Reference)
**Random effects model**			
CS	0.3 (0.5) / 391		−4.04 (−5.13 ; −2.95)
DHS	2.0 (1.6) / 21		−3.15 (−5.20 ; −1.10)
HA	2.6 (1.7) / 696		−1.15 (−1.79 ; −0.50)
THA	4.0 (2.0) / 383		0.00 (Reference)
Heterogeneity: $I^2 = 98\%$, $t^2 = 0.8$, $X^2(7) = 185.09$, $p < 0.001$			
Consistency: $X^2(1) = 152.28$, $p < 0.001$			

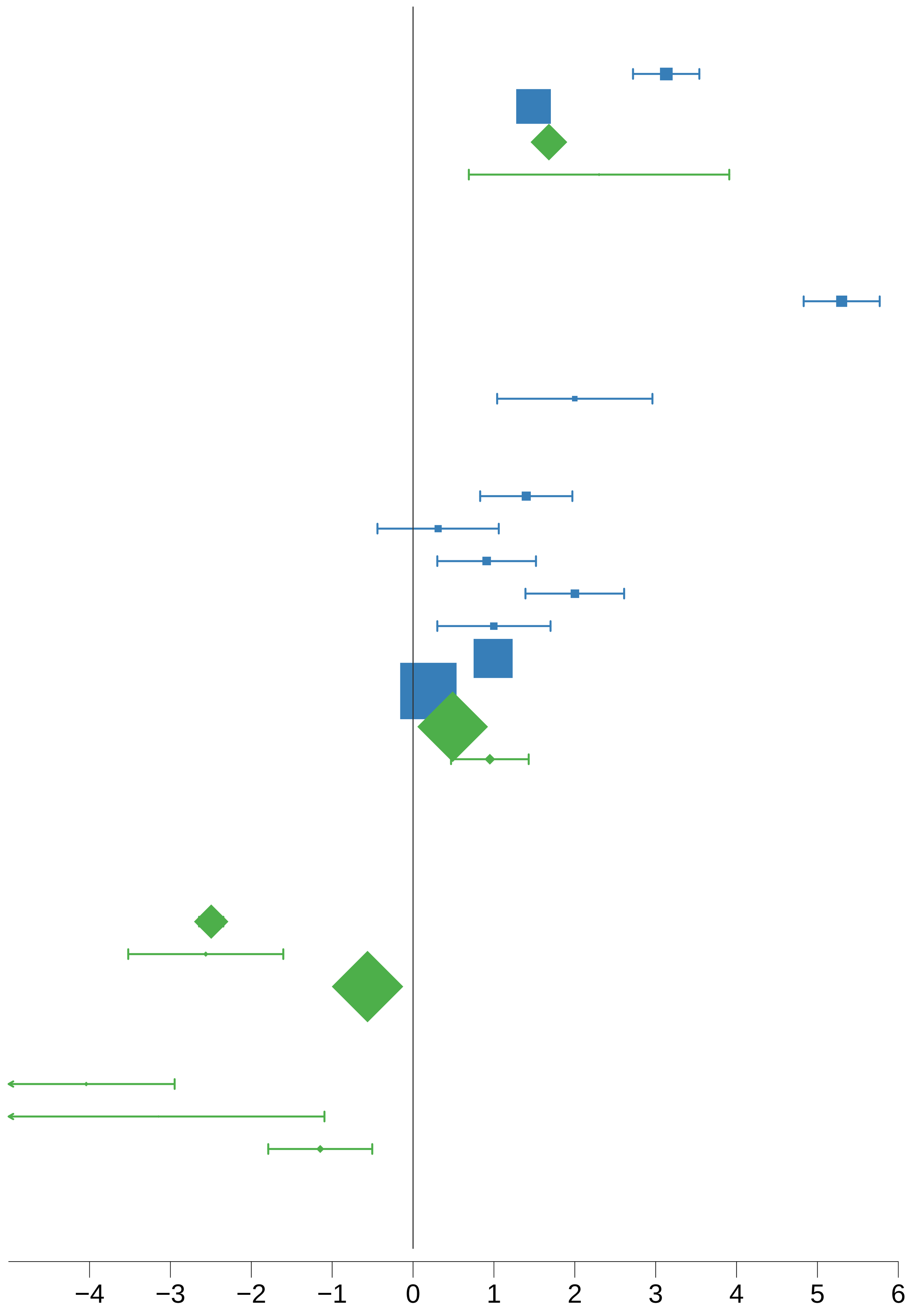

Supplement: Supplementary file 4 — Additional file 4: Forest plot of intraoperative blood loss (displaced femoral neck fractures only). Results are shown for a unit of 100 ml. CS, cannulated screw; DHS, dynamic hip screw; HA, hemiarthroplasty; THA, total hip arthroplasty; SD, standard deviation; MD, mean difference; CI, confidence interval. [file 13018_2023_4114_MOESM4_ESM.pdf]
