# Supplementary material for: Cannulated screws versus dynamic hip screw versus hemiarthroplasty versus total hip arthroplasty in patients with displaced and non-displaced femoral neck fractures: a systematic review and frequentist network meta-analysis of 5703 patients
Source: J Orthop Surg Res. 2023 Aug 26;18:625. doi: 10.1186/s13018-023-04114-8 (PMC10464356; doi:10.1186/s13018-023-04114-8)

	Mean (SD) / Patients	Mean (SD) / Patients	MD (95% CI)
HA vs CS			
Frihagen et al. 2007	0.6 (0.3) / 110	0.5 (0.3) / 112	0.08 (0.00 ; 0.16)
THA vs HA			
Chammout et al. 2019	0.7 (0.3) / 60	0.7 (0.2) / 60	−0.02 (−0.11 ; 0.07)
Hedbeck et al. 2011	0.7 (0.3) / 60	0.6 (0.3) / 60	0.05 (−0.06 ; 0.16)
**Fixed effects model**	**0.7 (0.3) / 120**	**0.6 (0.3) / 120**	**0.01 (−0.06 ; 0.08)**
**Random effects model**	**0.7 (0.3) / 120**	**0.6 (0.3) / 120**	**0.01 (−0.06 ; 0.08)**
Heterogeneity: $I^2 = 0$ %, $t^2 = 0.0$, $X^2 (1) = 0.97$, $p = 0.325$			
NETWORK META−ANALYSIS			
**Fixed effects model**			
CS	0.5 (0.3) / 112		−0.09 (−0.19 ; 0.01)
HA	0.6 (0.3) / 230		−0.01 (−0.08 ; 0.06)
THA	0.7 (0.3) / 120		0.00 (Reference)
**Random effects model**			
CS	0.5 (0.3) / 112		−0.09 (−0.19 ; 0.01)
HA	0.6 (0.3) / 230		−0.01 (−0.08 ; 0.06)
THA	0.7 (0.3) / 120		0.00 (Reference)
Heterogeneity: $I^2 = 0$ %, $t^2 = 0.0$, $X^2 (1) = 0.97$, $p = 0.325$			
Consistency: $X^2 (0) = 0.00$, $p = NA$			

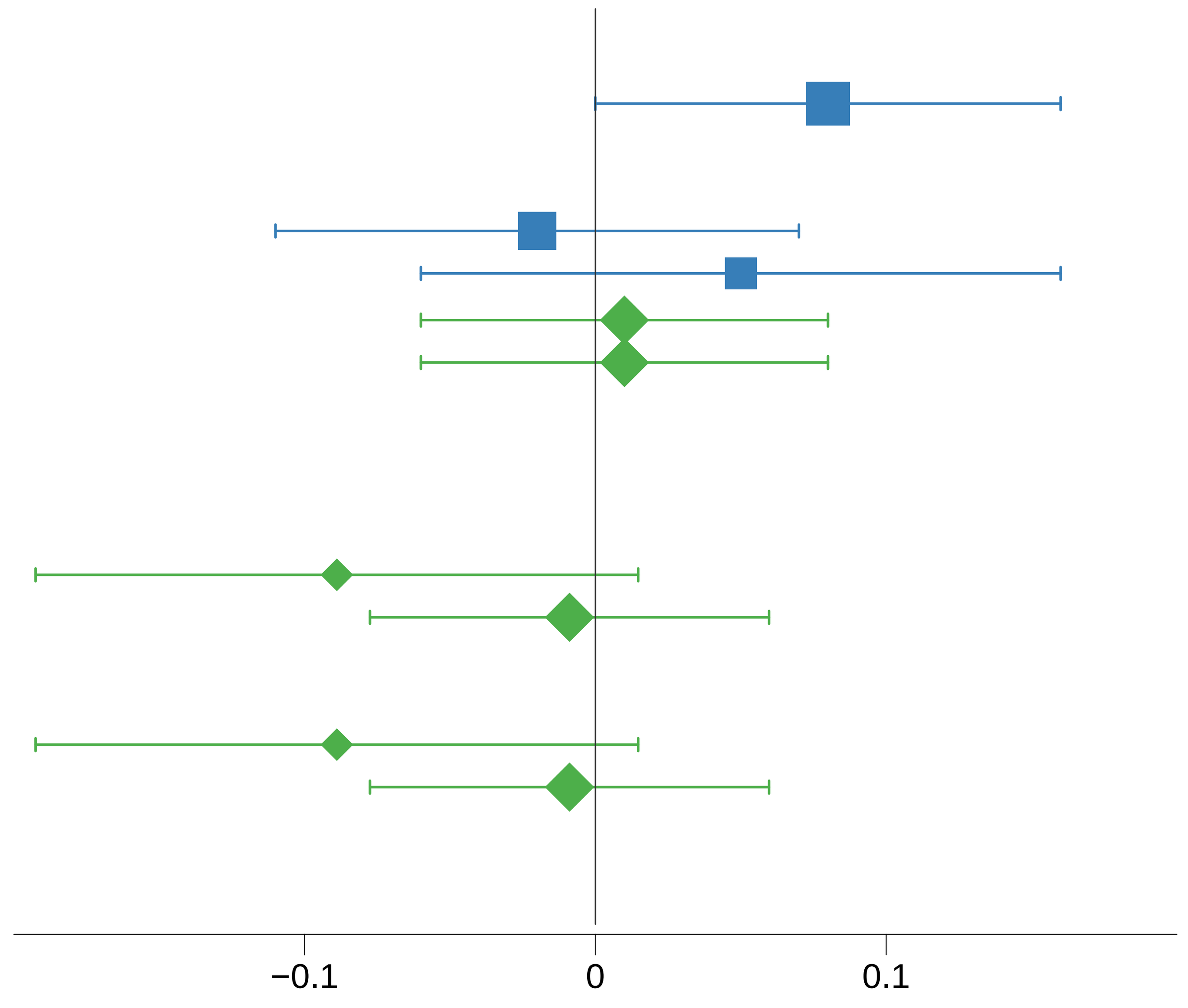

Supplement: Supplementary file 5 — Additional file 5: Forest plot of EQ 5D 3-4 months postoperatively (displaced femoral neck fractures only). CS, cannulated screw; HA, hemiarthroplasty; THA, total hip arthroplasty; SD, standard deviation; MD, mean difference; CI, confidence interval. [file 13018_2023_4114_MOESM5_ESM.pdf]
