# Supplementary material for: Cannulated screws versus dynamic hip screw versus hemiarthroplasty versus total hip arthroplasty in patients with displaced and non-displaced femoral neck fractures: a systematic review and frequentist network meta-analysis of 5703 patients
Source: J Orthop Surg Res. 2023 Aug 26;18:625. doi: 10.1186/s13018-023-04114-8 (PMC10464356; doi:10.1186/s13018-023-04114-8)

	Mean (SD) / Patients	Mean (SD) / Patients	MD (95% CI)
HA vs CS			
Frihagen et al. 2007	0.7 (0.2) / 110	0.6 (0.3) / 112	0.11 (0.04 ; 0.18)
THA vs HA			
Chammout et al. 2019	0.7 (0.3) / 60	0.6 (0.4) / 60	0.11 (0.00 ; 0.22)
Hedbeck et al. 2011	0.7 (0.3) / 60	0.6 (0.3) / 60	0.08 (−0.02 ; 0.18)
**Fixed effects model**	**0.7 (0.3) / 120**	**0.6 (0.3) / 120**	**0.09 (0.02 ; 0.17)**
**Random effects model**	**0.7 (0.3) / 120**	**0.6 (0.3) / 120**	**0.09 (0.02 ; 0.17)**
Heterogeneity: I² = 0 %, t² = 0.0 , X² (1) = 0.15 , p = 0.695			
NETWORK META−ANALYSIS			
**Fixed effects model**			
CS	0.6 (0.3) / 112		−0.20 (−0.31 ; −0.10)
HA	0.6 (0.3) / 230		−0.09 (−0.17 ; −0.02)
THA	0.7 (0.3) / 120		0.00 (Reference)
**Random effects model**			
CS	0.6 (0.3) / 112		−0.20 (−0.31 ; −0.10)
HA	0.6 (0.3) / 230		−0.09 (−0.17 ; −0.02)
THA	0.7 (0.3) / 120		0.00 (Reference)
Heterogeneity: I² = 0 %, t² = 0.0 , X² (1) = 0.15 , p = 0.695			
Consistency: X² (0) = 0.00 , p = NA			

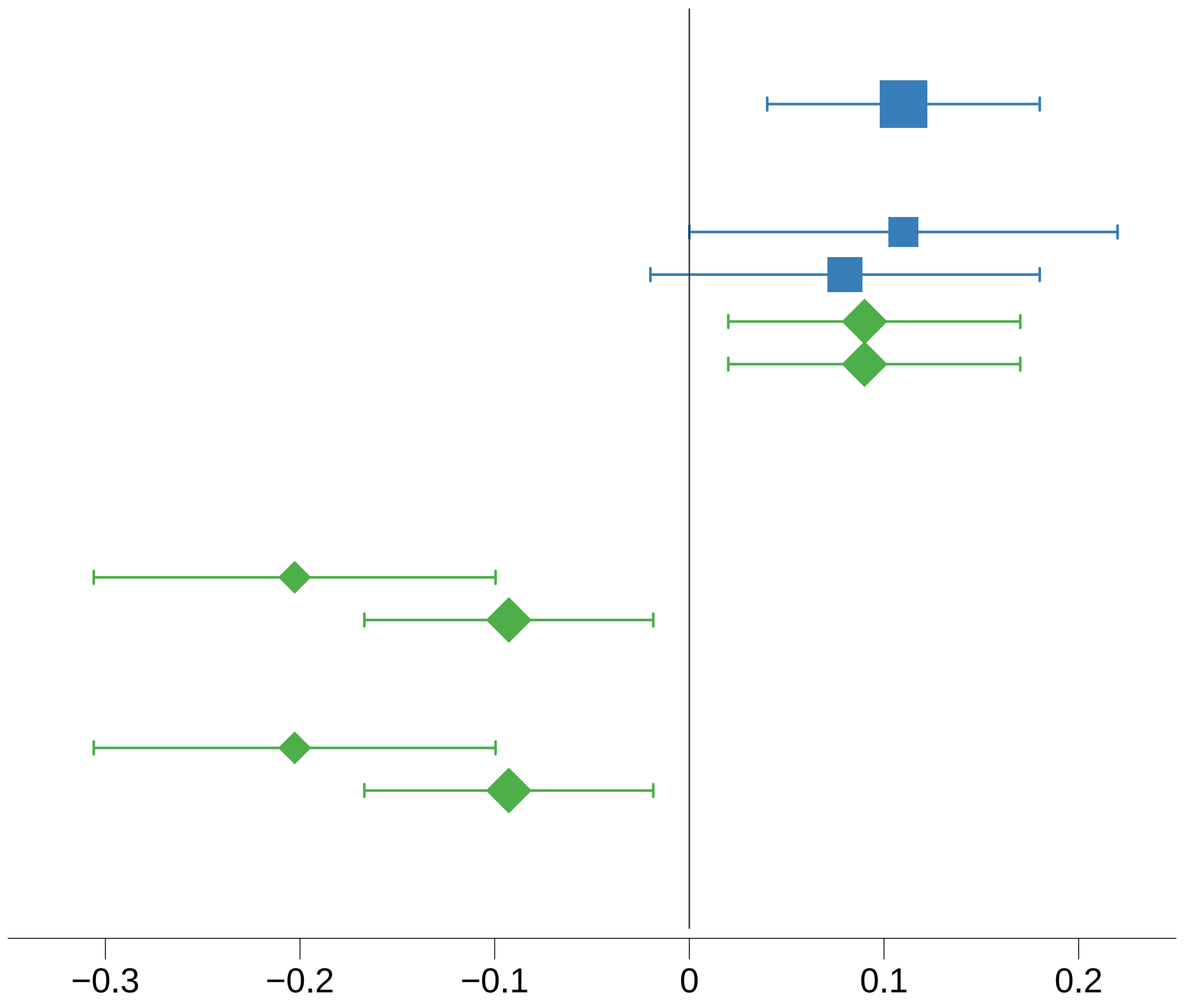

Supplement: Supplementary file 7 — Additional file 7: Forest plot of EQ 5D 2 years postoperatively (displaced femoral neck fractures only). CS, cannulated screw; HA, hemiarthroplasty; THA, total hip arthroplasty; SD, standard deviation; MD, mean difference; CI, confidence interval. [file 13018_2023_4114_MOESM7_ESM.pdf]
