# Supplementary material for: Cannulated screws versus dynamic hip screw versus hemiarthroplasty versus total hip arthroplasty in patients with displaced and non-displaced femoral neck fractures: a systematic review and frequentist network meta-analysis of 5703 patients
Source: J Orthop Surg Res. 2023 Aug 26;18:625. doi: 10.1186/s13018-023-04114-8 (PMC10464356; doi:10.1186/s13018-023-04114-8)

	Mean (SD) / Patients	Mean (SD) / Patients	MD (95% CI)
HA vs CS			
Frihagen et al. 2007	67.7 (15.8) / 110	59.6 (19.5) / 112	8.10 (3.44 ; 12.76)
Steon et al. 2014	67.0 (15.8) / 110	59.0 (19.5) / 112	8.00 (3.34 ; 12.66)
**Fixed effects model**	**67.3 (15.8) / 220**	**59.3 (19.5) / 224**	**8.05 (4.75 ; 11.35)**
**Random effects model**	**67.3 (15.8) / 220**	**59.3 (19.5) / 224**	**8.05 (4.75 ; 11.35)**
Heterogeneity: I² = 0 %, t² = 0.0 , X² (1) = 0.00 , p = 0.976			
HA vs DHS			
Mouzopoulos et al. 2008	58.3 (6.2) / 34	58.1 (5.3) / 38	0.20 (−3.07 ; 3.47)
THA vs DHS			
Mouzopoulos et al. 2008	59.4 (5.8) / 37	58.1 (5.3) / 38	1.30 (−1.64 ; 4.24)
THA vs HA			
Cadossi et al. 2013	74.0 (14.6) / 60	72.3 (16.2) / 60	1.70 (−3.81 ; 7.21)
Chammout et al. 2019	70.0 (13.0) / 60	69.0 (14.0) / 60	1.00 (−3.83 ; 5.83)
Mouzopoulos et al. 2008	59.4 (5.8) / 37	58.3 (6.2) / 34	1.10 (−2.53 ; 4.73)
Ukaj et al. 2019	88.0 (5.7) / 47	86.8 (6.5) / 49	1.14 (−1.29 ; 3.57)
Blomfeldt et al. 2007	82.5 (11.5) / 60	77.5 (15.7) / 60	5.00 (0.08 ; 9.92)
Hedbeck et al. 2011	82.5 (11.5) / 60	77.5 (12.4) / 60	5.00 (0.72 ; 9.28)
Macaulay et al. 2007	76.1 (26.5) / 17	77.1 (12.0) / 23	−1.00 (−14.52 ; 12.52)
Ukaj et al. 2019	90.9 (6.9) / 47	87.3 (8.7) / 49	3.60 (0.47 ; 6.73)
**Fixed effects model**	**78.4 (15.0) / 388**	**76.1 (14.9) / 395**	**2.32 (0.94 ; 3.70)**
**Random effects model**	**78.4 (15.0) / 388**	**76.1 (14.9) / 395**	**2.32 (0.94 ; 3.70)**
Heterogeneity: I² = 0 %, t² = 0.0 , X² (7) = 5.46 , p = 0.603			
NETWORK META−ANALYSIS			
**Fixed effects model**			
CS	59.3 (19.5) / 224		−10.26 (−13.81 ; −6.71)
DHS	58.1 (5.3) / 76		−1.80 (−4.06 ; 0.47)
HA	73.0 (15.8) / 649		−2.21 (−3.53 ; −0.89)
THA	78.4 (15.0) / 425		0.00 (Reference)
**Random effects model**			
CS	59.3 (19.5) / 224		−10.26 (−13.81 ; −6.71)
DHS	58.1 (5.3) / 76		−1.80 (−4.06 ; 0.47)
HA	73.0 (15.8) / 649		−2.21 (−3.53 ; −0.89)
THA	78.4 (15.0) / 425		0.00 (Reference)
Heterogeneity: I² = 0 %, t² = 0.0 , X² (7) = 4.69 , p = 0.698			
Consistency: X² (1) = 0.78 , p = 0.378			

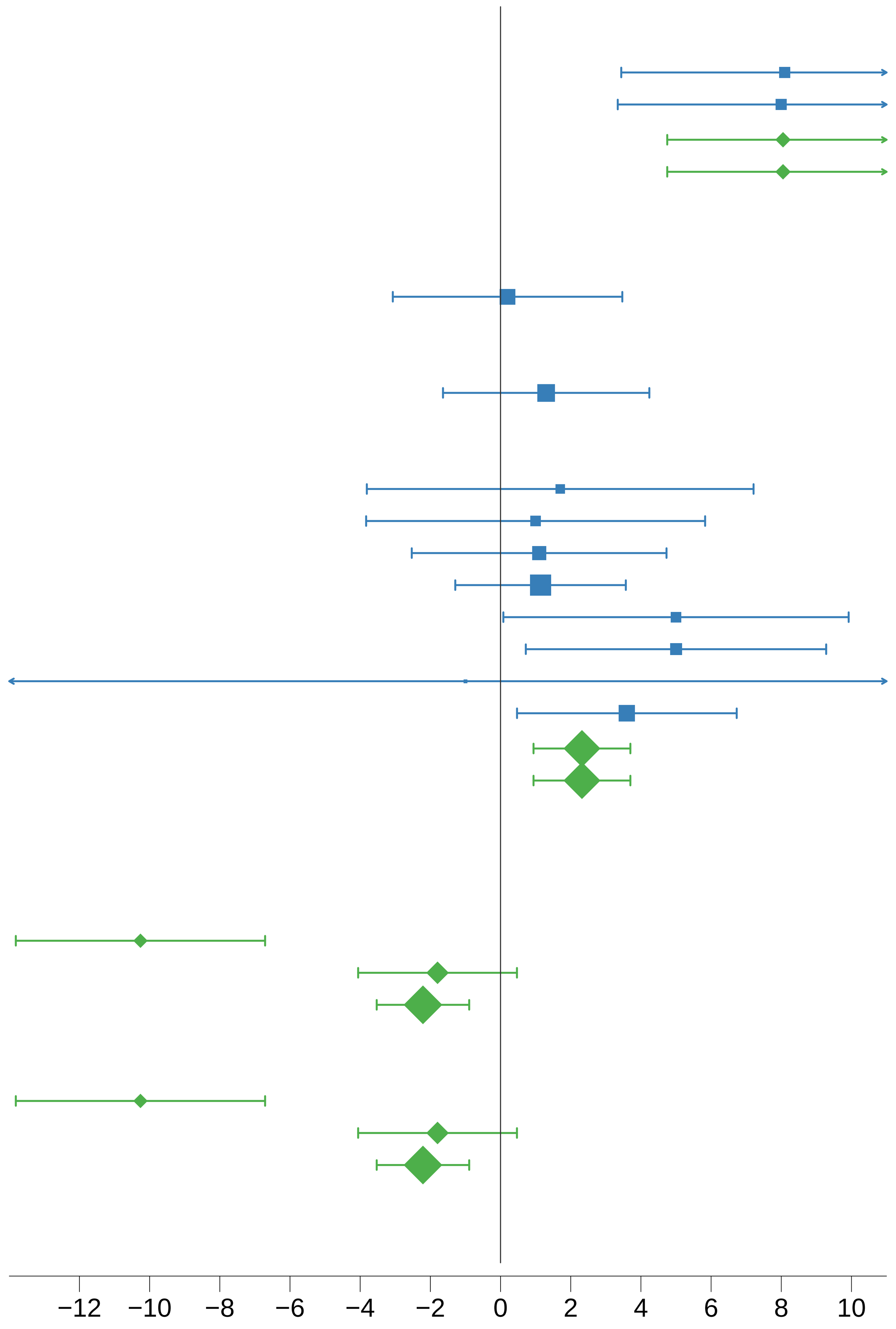

Supplement: Supplementary file 8 — Additional file 8: Forest plot of Harris Hip Score ≤ 6 months postoperatively (displaced femoral neck fractures only). CS, cannulated screw; DHS, dynamic hip screw; HA, hemiarthroplasty; THA, total hip arthroplasty; SD, standard deviation; MD, mean difference; CI, confidence interval. [file 13018_2023_4114_MOESM8_ESM.pdf]
