# Supplementary material for: Cannulated screws versus dynamic hip screw versus hemiarthroplasty versus total hip arthroplasty in patients with displaced and non-displaced femoral neck fractures: a systematic review and frequentist network meta-analysis of 5703 patients
Source: J Orthop Surg Res. 2023 Aug 26;18:625. doi: 10.1186/s13018-023-04114-8 (PMC10464356; doi:10.1186/s13018-023-04114-8)

	Mean (SD) / Patients	Mean (SD) / Patients	MD (95% CI)
HA vs CS			
Frihagen et al. 2007	72.6 (17.5) / 110	65.8 (15.9) / 112	6.80 (2.40 ; 11.20)
Steon et al. 2014	72.0 (17.5) / 110	65.0 (15.9) / 112	7.00 (2.60 ; 11.40)
**Fixed effects model**	**72.3 (17.5) / 220**	**65.4 (15.9) / 224**	**6.90 (3.79 ; 10.01)**
**Random effects model**	**72.3 (17.5) / 220**	**65.4 (15.9) / 224**	**6.90 (3.79 ; 10.01)**
Heterogeneity: I² = 0 %, t² = 0.0 , X² (1) = 0.00 , p = 0.950			
HA vs DHS			
Davison et al. 2001	72.2 (16.5) / 187	70.8 (5.3) / 93	1.40 (−1.20 ; 4.00)
Mouzopoulos et al. 2008	77.8 (9.6) / 34	71.3 (5.3) / 38	6.51 (1.50 ; 11.52)
**Fixed effects model**	**73.1 (15.7) / 221**	**70.9 (5.3) / 131**	**2.48 (0.18 ; 4.79)**
**Random effects model**	**73.1 (15.7) / 221**	**70.9 (5.3) / 131**	**3.49 (−1.44 ; 8.41)**
Heterogeneity: I² = 80 %, t² = 10.5 , X² (1) = 5.02 , p = 0.025			
THA vs DHS			
Mouzopoulos et al. 2008	81.6 (4.9) / 37	71.3 (5.3) / 38	10.30 (7.85 ; 12.75)
THA vs HA			
Blomfeldt et al. 2007	87.2 (9.1) / 60	79.4 (16.5) / 60	7.80 (3.04 ; 12.56)
Cadossi et al. 2013	73.1 (9.1) / 47	74.7 (16.5) / 49	−1.60 (−6.90 ; 3.70)
Chammout et al. 2019	74.0 (9.1) / 60	71.0 (16.5) / 60	3.00 (−1.76 ; 7.76)
Hedbeck et al. 2011	87.2 (9.4) / 60	79.4 (12.3) / 60	7.80 (3.88 ; 11.72)
Macaulay et al. 2007	84.2 (12.0) / 17	80.6 (14.3) / 23	3.60 (−4.57 ; 11.77)
Mouzopoulos et al. 2008	81.6 (4.9) / 37	77.8 (9.6) / 34	3.79 (−0.91 ; 8.49)
Sharma et al. 2016	90.0 (9.1) / 40	80.0 (16.5) / 40	10.00 (4.16 ; 15.84)
Ukaj et al. 2019	92.3 (7.2) / 47	88.4 (8.3) / 49	3.85 (0.75 ; 6.95)
van den Bekerom et al. 2010	76.0 (9.1) / 115	73.9 (16.5) / 137	2.10 (−1.12 ; 5.32)
**Fixed effects model**	**81.7 (11.1) / 483**	**77.4 (15.6) / 512**	**4.28 (2.83 ; 5.72)**
**Random effects model**	**81.7 (11.1) / 483**	**77.4 (15.6) / 512**	**4.42 (2.29 ; 6.54)**
Heterogeneity: I² = 49 %, t² = 4.6 , X² (8) = 15.82 , p = 0.045			
NETWORK META−ANALYSIS			
**Fixed effects model**			
CS	65.4 (15.9) / 224		−11.73 (−15.11 ; −8.35)
DHS	70.9 (5.3) / 169		−8.71 (−10.53 ; −6.89)
HA	75.1 (16.4) / 953		−4.83 (−6.16 ; −3.50)
THA	81.7 (11.1) / 520		0.00 (Reference)
**Random effects model**			
CS	65.4 (15.9) / 224		−11.57 (−16.45 ; −6.69)
DHS	70.9 (5.3) / 169		−8.65 (−12.30 ; −5.00)
HA	75.1 (16.4) / 953		−4.67 (−6.74 ; −2.60)
THA	81.7 (11.1) / 520		0.00 (Reference)
Heterogeneity: I² = 56 %, t² = 5.1 , X² (8) = 15.75 , p = 0.046			
Consistency: X² (2) = 6.78 , p = 0.034			

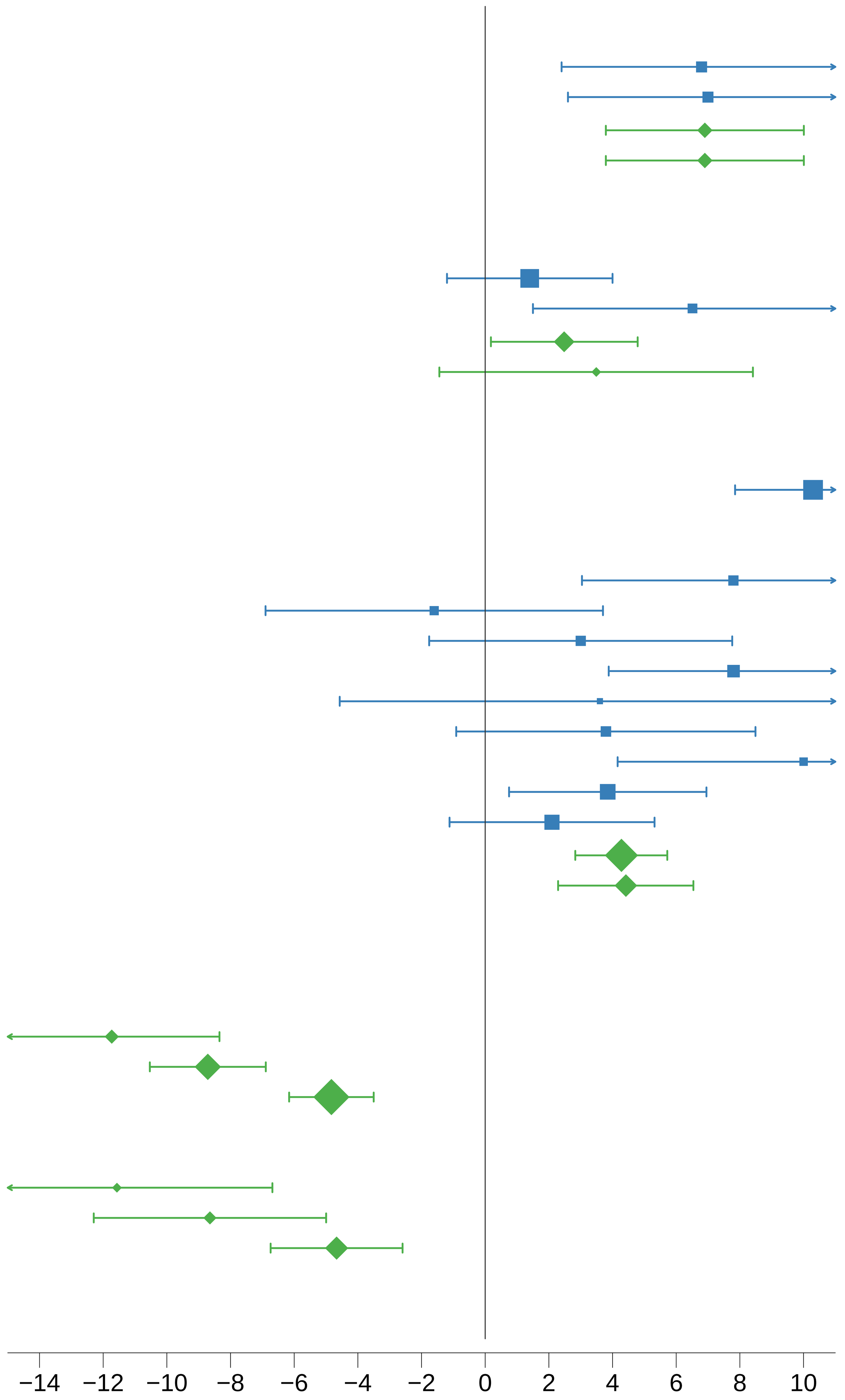

Supplement: Supplementary file 9 — Additional file 9: Forest plot of Harris Hip Score 12 months postoperatively (displaced femoral neck fractures only). CS, cannulated screw; DHS, dynamic hip screw; HA, hemiarthroplasty; THA, total hip arthroplasty; SD, standard deviation; MD, mean difference; CI, confidence interval. [file 13018_2023_4114_MOESM9_ESM.pdf]
