# Supplementary material for: Cannulated screws versus dynamic hip screw versus hemiarthroplasty versus total hip arthroplasty in patients with displaced and non-displaced femoral neck fractures: a systematic review and frequentist network meta-analysis of 5703 patients
Source: J Orthop Surg Res. 2023 Aug 26;18:625. doi: 10.1186/s13018-023-04114-8 (PMC10464356; doi:10.1186/s13018-023-04114-8)

	Mean (SD) / Patients	Mean (SD) / Patients	MD (95% CI)
HA vs CS			
Frihagen et al. 2007	70.6 (19.1) / 110	67.3 (15.5) / 112	3.30 (−1.28 ; 7.88)
Steon et al. 2014	70.0 (19.1) / 110	67.0 (15.5) / 112	3.00 (−1.58 ; 7.58)
**Fixed effects model**	**70.3 (19.1) / 220**	**67.1 (15.5) / 224**	**3.15 (−0.09 ; 6.39)**
**Random effects model**	**70.3 (19.1) / 220**	**67.1 (15.5) / 224**	**3.15 (−0.09 ; 6.39)**
Heterogeneity: I² = 0 %, t² = 0.0 , X² (1) = 0.01 , p = 0.928			
HA vs DHS			
Davison et al. 2001	74.3 (16.8) / 187	70.7 (16.6) / 93	3.60 (−0.54 ; 7.74)
THA vs HA			
Cadossi et al. 2013	71.9 (13.1) / 47	75.0 (16.8) / 49	−3.10 (−9.11 ; 2.91)
Chammout et al. 2019	76.0 (15.0) / 60	74.0 (14.0) / 60	2.00 (−3.19 ; 7.19)
Hedbeck et al. 2011	87.2 (10.1) / 60	77.9 (12.5) / 60	9.30 (5.23 ; 13.37)
Macaulay et al. 2008	84.0 (12.2) / 17	81.1 (11.7) / 23	2.90 (−4.62 ; 10.42)
Sonaje et al. 2018	88.0 (5.8) / 21	83.9 (6.6) / 21	4.10 (0.34 ; 7.86)
**Fixed effects model**	**80.2 (13.8) / 205**	**77.1 (13.8) / 213**	**4.17 (2.01 ; 6.34)**
**Random effects model**	**80.2 (13.8) / 205**	**77.1 (13.8) / 213**	**3.41 (−0.60 ; 7.41)**
Heterogeneity: I² = 68 %, t² = 13.7 , X² (4) = 12.52 , p = 0.014			
NETWORK META−ANALYSIS			
**Fixed effects model**			
CS	67.1 (15.5) / 224		−7.32 (−11.22 ; −3.43)
DHS	70.7 (16.6) / 93		−7.77 (−12.45 ; −3.10)
HA	73.8 (16.9) / 620		−4.17 (−6.34 ; −2.01)
THA	80.2 (13.8) / 205		0.00 (Reference)
**Random effects model**			
CS	67.1 (15.5) / 224		−6.66 (−13.06 ; −0.25)
DHS	70.7 (16.6) / 93		−7.11 (−15.21 ; 1.00)
HA	73.8 (16.9) / 620		−3.51 (−7.05 ; 0.04)
THA	80.2 (13.8) / 205		0.00 (Reference)
Heterogeneity: I² = 60 %, t² = 9.4 , X² (5) = 12.53 , p = 0.028			
Consistency: X² (0) = 0.00 , p = NA			

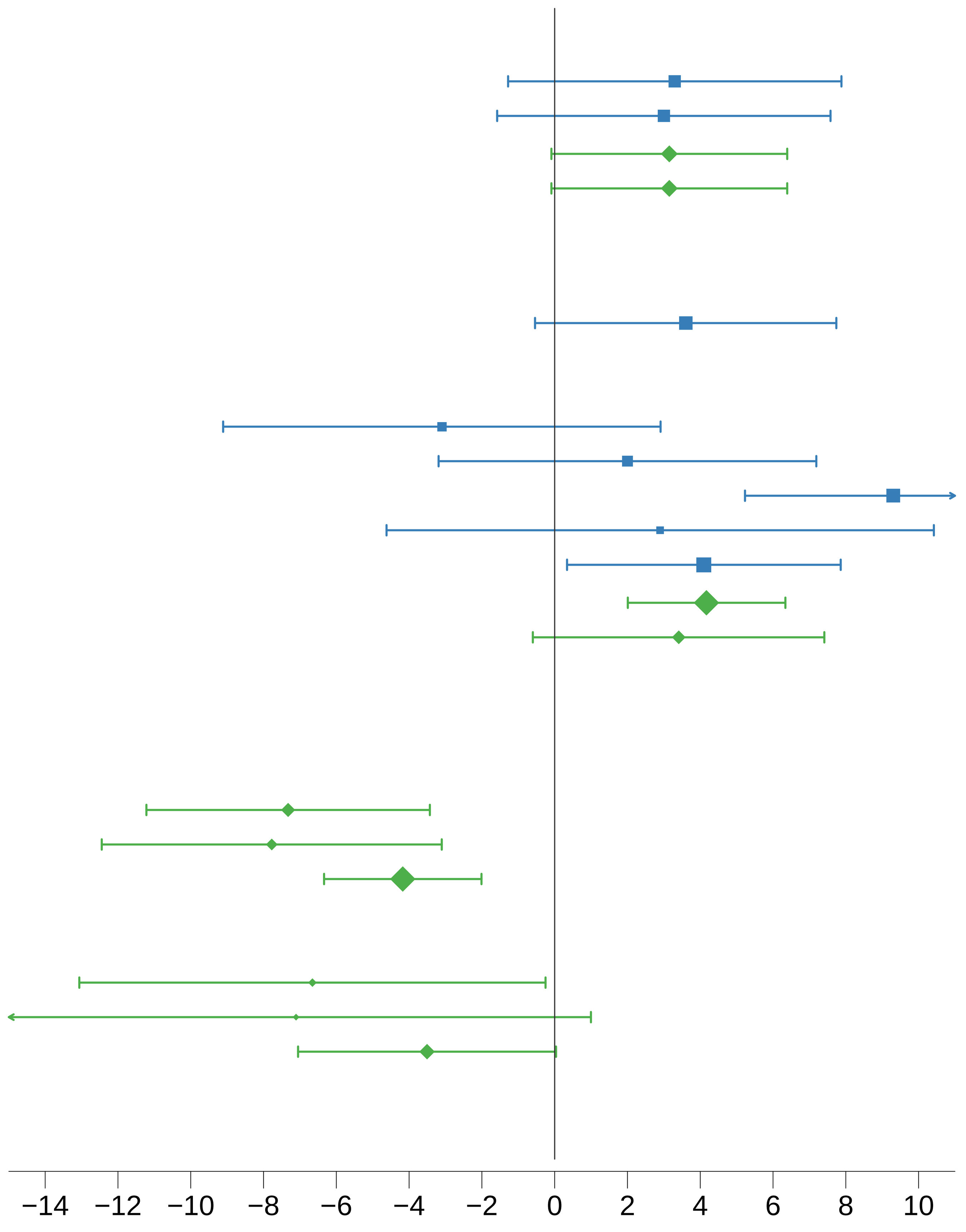

Supplement: Supplementary file 10 — Additional file 10: Forest plot of Harris Hip Score 2 years postoperatively (displaced femoral neck fractures only). CS, cannulated screw; DHS, dynamic hip screw; HA, hemiarthroplasty; THA, total hip arthroplasty; SD, standard deviation; MD, mean difference; CI, confidence interval. [file 13018_2023_4114_MOESM10_ESM.pdf]
