# Supplementary material for: Cannulated screws versus dynamic hip screw versus hemiarthroplasty versus total hip arthroplasty in patients with displaced and non-displaced femoral neck fractures: a systematic review and frequentist network meta-analysis of 5703 patients
Source: J Orthop Surg Res. 2023 Aug 26;18:625. doi: 10.1186/s13018-023-04114-8 (PMC10464356; doi:10.1186/s13018-023-04114-8)

	Mean (SD) / Patients	Mean (SD) / Patients	MD (95% CI)
HA vs DHS			
Davison et al. 2001	73.6 (10.1) / 187	70.6 (9.9) / 93	3.00 (0.53 ; 5.47)
Davison et al. 2001	73.1 (12.7) / 187	71.8 (6.7) / 93	1.30 (−0.98 ; 3.58)
Mouzopoulos et al. 2008	79.5 (6.5) / 34	73.6 (6.7) / 38	5.90 (2.13 ; 9.67)
Davison et al. 2001	72.7 (5.4) / 187	70.0 (5.9) / 93	2.70 (1.27 ; 4.13)
**Fixed effects model**	**73.5 (9.8) / 595**	**71.1 (7.6) / 317**	**2.70 (1.66 ; 3.75)**
**Random effects model**	**73.5 (9.8) / 595**	**71.1 (7.6) / 317**	**2.77 (1.43 ; 4.12)**
Heterogeneity: I^2 = 47 %, t^2 = 1.1 , X^2 (3) = 5.67 , p = 0.129			
THA vs DHS			
Mouzopoulos et al. 2008	83.7 (6.5) / 37	73.6 (6.7) / 38	10.10 (6.48 ; 13.72)
THA vs HA			
Cadossi et al. 2013	71.3 (6.0) / 47	78.7 (10.1) / 49	−7.40 (−10.70 ; −4.10)
Ukaj et al. 2019	92.5 (6.0) / 47	88.3 (7.7) / 49	4.20 (1.44 ; 6.96)
Hedbeck et al. 2011	89.0 (8.1) / 60	75.2 (15.4) / 60	13.80 (9.40 ; 18.20)
Mouzopoulos et al. 2008	83.7 (6.5) / 37	79.5 (6.5) / 34	4.20 (0.49 ; 7.91)
van den Bekerom et al. 2010	75.2 (5.9) / 115	71.9 (5.4) / 137	3.30 (1.89 ; 4.71)
**Fixed effects model**	**81.0 (10.1) / 306**	**76.7 (10.7) / 329**	**3.00 (1.91 ; 4.08)**
**Random effects model**	**81.0 (10.1) / 306**	**76.7 (10.7) / 329**	**3.49 (−1.50 ; 8.48)**
Heterogeneity: I^2 = 94 %, t^2 = 27.5 , X^2 (4) = 62.69 , p < 0.001			
NETWORK META−ANALYSIS			
**Fixed effects model**			
DHS	71.1 (7.6) / 355		−6.35 (−7.75 ; −4.96)
HA	74.5 (10.3) / 924		−3.34 (−4.38 ; −2.29)
THA	81.0 (10.1) / 343		0.00 (Reference)
**Random effects model**			
DHS	71.1 (7.6) / 355		−7.19 (−11.95 ; −2.44)
HA	74.5 (10.3) / 924		−3.75 (−7.23 ; −0.27)
THA	81.0 (10.1) / 343		0.00 (Reference)
Heterogeneity: I^2 = 90 %, t^2 = 14.1 , X^2 (5) = 63.34 , p < 0.001			
Consistency: X^2 (2) = 8.25 , p = 0.016			

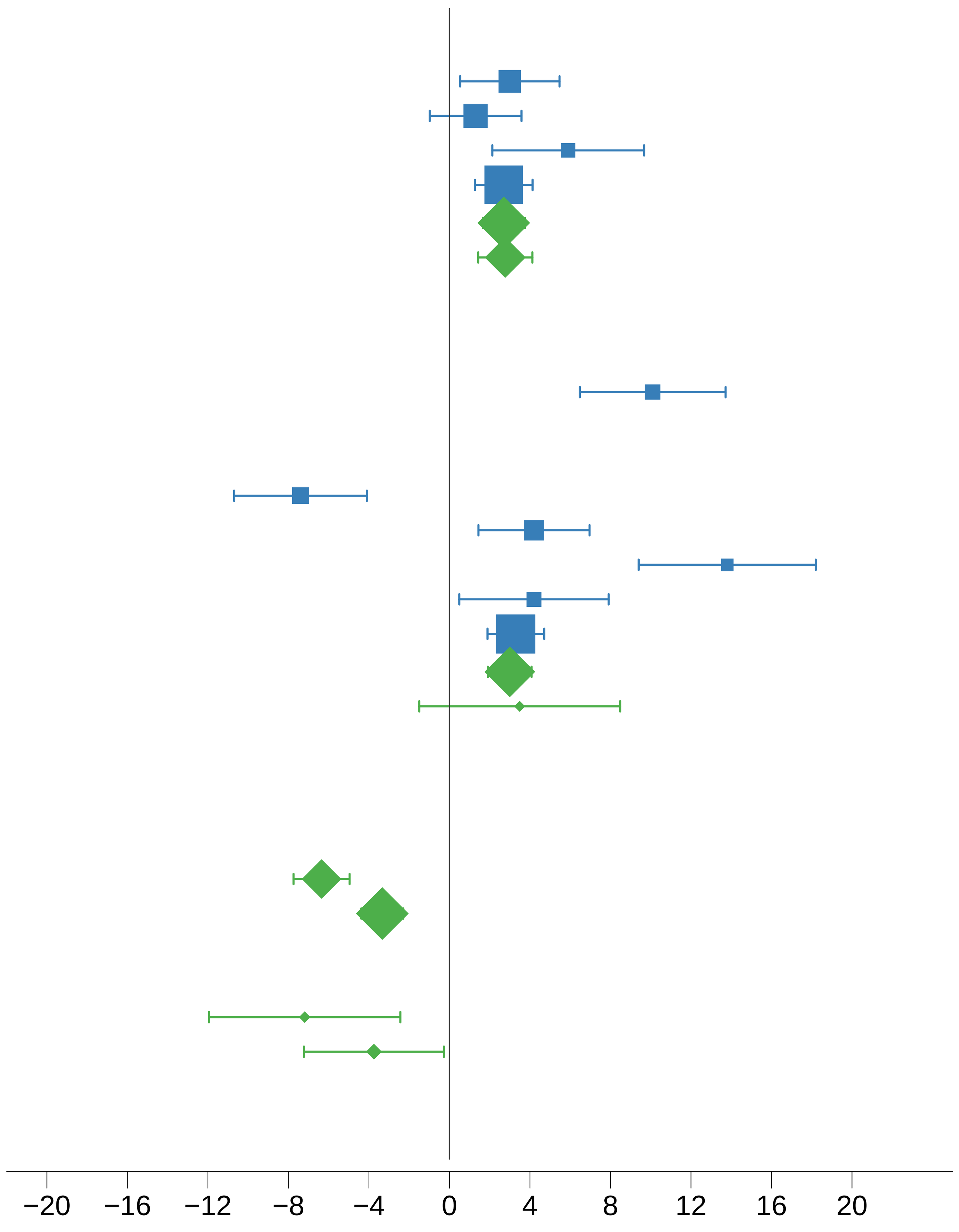

Supplement: Supplementary file 11 — Additional file 11: Forest plot of Harris Hip Score 3-5 years postoperatively (displaced femoral neck fractures only). CS, cannulated screw; DHS, dynamic hip screw; HA, hemiarthroplasty; THA, total hip arthroplasty; SD, standard deviation; MD, mean difference; CI, confidence interval. [file 13018_2023_4114_MOESM11_ESM.pdf]
