# Supplementary material for: Cannulated screws versus dynamic hip screw versus hemiarthroplasty versus total hip arthroplasty in patients with displaced and non-displaced femoral neck fractures: a systematic review and frequentist network meta-analysis of 5703 patients
Source: J Orthop Surg Res. 2023 Aug 26;18:625. doi: 10.1186/s13018-023-04114-8 (PMC10464356; doi:10.1186/s13018-023-04114-8)

	Mean (SD) / Patients	Mean (SD) / Patients	MD (95% CI)
HA vs CS			
Frihagen et al. 2007	10.2 (11.9) / 110	8.2 (7.4) / 112	2.00 (−0.62 ; 4.62)
Roden et al. 2003	15.0 (8.3) / 47	15.0 (5.6) / 53	0.00 (−2.80 ; 2.80)
**Fixed effects model**	**11.6 (11.2) / 157**	**10.4 (7.6) / 165**	**1.07 (−0.85 ; 2.98)**
**Random effects model**	**11.6 (11.2) / 157**	**10.4 (7.6) / 165**	**1.07 (−0.89 ; 3.02)**
Heterogeneity: $I^2 = 4$ %, $t^2 = 0.1$, X^2 (1) = 1.04 , p = 0.307			
THA vs CS			
Jonsson et al. 1996	15.0 (5.9) / 25	12.0 (5.6) / 25	3.00 (−0.20 ; 6.20)
HA vs DHS			
Davison et al. 2001	15.0 (8.3) / 187	14.0 (2.8) / 93	1.00 (−0.31 ; 2.31)
Mouzopoulos et al. 2008	9.1 (3.4) / 34	13.0 (2.8) / 38	−3.90 (−5.44 ; −2.36)
**Fixed effects model**	**14.1 (8.0) / 221**	**13.7 (2.8) / 131**	**−1.07 (−2.07 ; −0.07)**
**Random effects model**	**14.1 (8.0) / 221**	**13.7 (2.8) / 131**	**−1.43 (−6.24 ; 3.37)**
Heterogeneity: $I^2 = 96$ %, $t^2 = 11.5$, X^2 (1) = 24.10 , p < 0.001			
THA vs DHS			
Mouzopoulos et al. 2008	8.3 (6.2) / 37	13.0 (2.8) / 38	−4.70 (−7.38 ; −2.02)
THA vs HA			
Cadossi et al. 2013	9.9 (5.9) / 47	8.7 (8.3) / 49	1.20 (−1.67 ; 4.07)
Iorio et al. 2019	6.1 (5.9) / 30	5.5 (8.3) / 30	0.60 (−3.04 ; 4.24)
Macaulay et al. 2007	7.7 (5.5) / 17	5.4 (2.8) / 23	2.30 (−0.55 ; 5.15)
Mouzopoulos et al. 2008	8.3 (6.2) / 37	9.1 (3.4) / 34	−0.80 (−4.25 ; 2.65)
Parker et al. 2019	14.5 (5.9) / 52	9.2 (8.3) / 53	5.30 (2.55 ; 8.05)
Schleicher et al. 2003	19.0 (5.9) / 54	20.5 (8.3) / 52	−1.50 (−4.25 ; 1.25)
Sharma et al. 2016	14.0 (5.9) / 40	14.0 (8.3) / 40	0.00 (−3.16 ; 3.16)
van den Bekerom et al. 2010	18.4 (5.9) / 115	17.1 (8.3) / 137	1.30 (−0.46 ; 3.06)
**Fixed effects model**	**14.1 (7.5) / 392**	**13.1 (9.2) / 418**	**1.22 (0.26 ; 2.17)**
**Random effects model**	**14.1 (7.5) / 392**	**13.1 (9.2) / 418**	**1.14 (−0.30 ; 2.58)**
Heterogeneity: $I^2 = 57$ %, $t^2 = 2.4$, X^2 (7) = 16.26 , p = 0.023			
NETWORK META−ANALYSIS			
**Fixed effects model**			
CS	10.6 (7.3) / 190		−2.16 (−3.93 ; −0.40)
DHS	13.7 (2.8) / 169		0.81 (−0.40 ; 2.03)
HA	13.3 (9.5) / 796		−0.80 (−1.68 ; 0.09)
THA	14.2 (7.4) / 454		0.00 (Reference)
**Random effects model**			
CS	10.6 (7.3) / 190		−2.26 (−5.67 ; 1.14)
DHS	13.7 (2.8) / 169		1.27 (−2.22 ; 4.76)
HA	13.3 (9.5) / 796		−0.91 (−2.74 ; 0.92)
THA	14.2 (7.4) / 454		0.00 (Reference)
Heterogeneity: $I^2 = 79$ %, $t^2 = 5.7$, X^2 (7) = 14.39 , p = 0.045			
Consistency: X^2 (3) = 34.26 , p < 0.001			

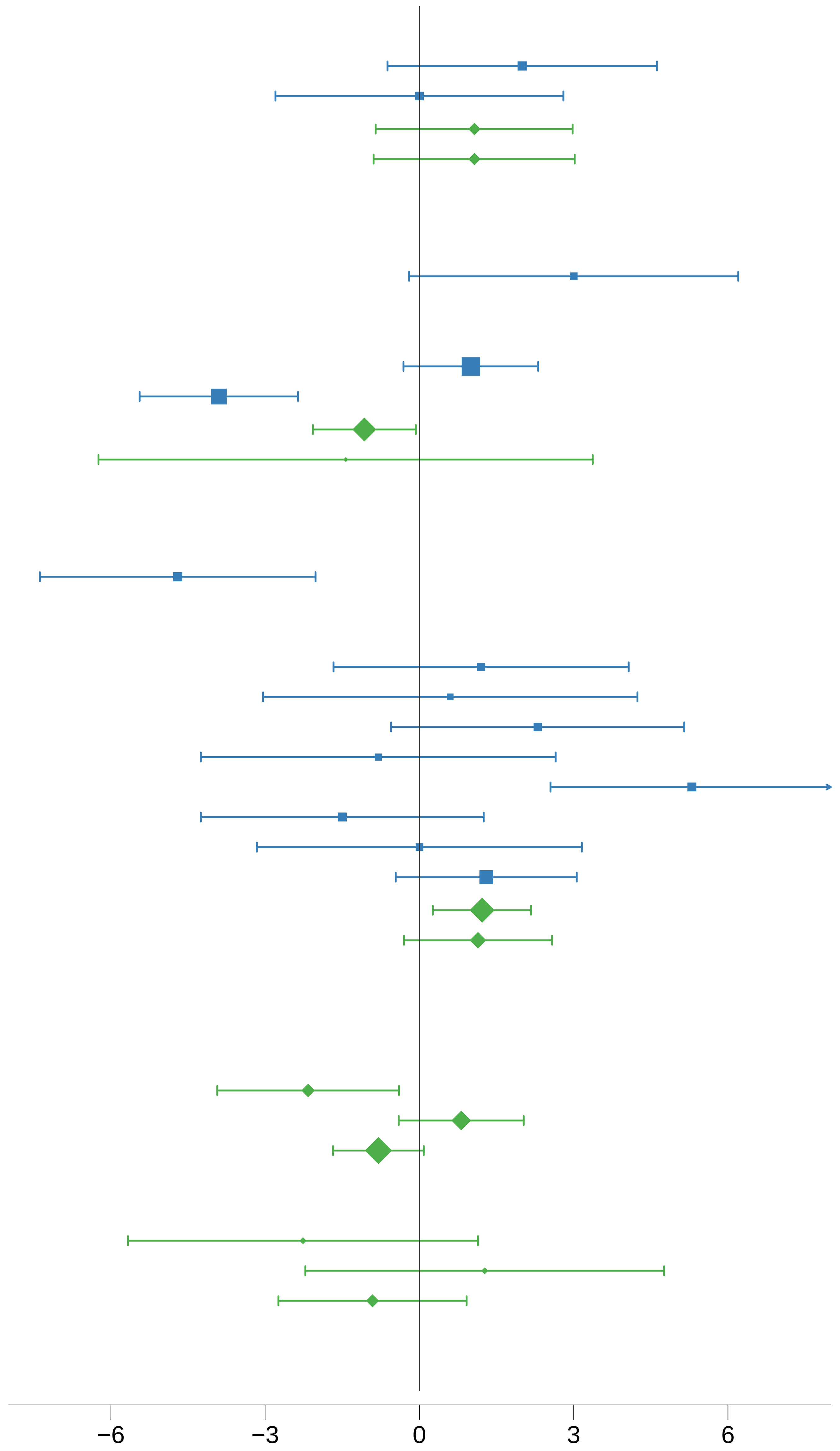

Supplement: Supplementary file 12 — Additional file 12: Forest plot of hospital stay (displaced femoral neck fractures only). CS, cannulated screw; DHS, dynamic hip screw; HA, hemiarthroplasty; THA, total hip arthroplasty; SD, standard deviation; MD, mean difference; CI, confidence interval. [file 13018_2023_4114_MOESM12_ESM.pdf]
